# A geochemical characterization of lead ores in China: An isotope database for provenancing archaeological materials

**DOI:** 10.1371/journal.pone.0215973

**Published:** 2019-04-24

**Authors:** Yiu-Kang Hsu, Benjamin J. Sabatini

**Affiliations:** 1 Deutsches Bergbau-Museum Bochum, Bochum, North Rhine-Westphalia, Germany; 2 USTC Archaeometry Laboratory, University of Science and Technology of China, Hefei, Anhui, China; 3 Department of Materials Science & Engineering, Massachusetts Institute of technology, Cambridge, Massachusetts, United States of America; Zhejiang University, CHINA

## Abstract

A well reasoned lead (Pb) isotope-driven provenance study lies in concert with a comprehensively evaluated database of geological ore sources and accompanying archaeological and contextual information. In this paper we have compiled and evaluated all currently available Pb isotope data for galena and K-feldspars in China, and provided geological interpretations for how their ore-forming substances evolved across relevant tectonic terrains. We pay particular attention to the geological settings of host ore deposits that were likely exploited in ancient and historic China, detailing the heterogeneity and homogeneity of their ore formation across different metallogenic provinces and belts. Using the isotope database, and supportive geological and archaeological background information, three case studies are presented that detail the provenancing of Chinese cultural materials. The isotope data themselves are presented in ternary diagrams that allow for their concise and accurate comparison.

## Introduction

Lead (Pb) isotope geochemistry has played a vital role in tracing the fluid pathways and sources of metal in ore deposits [1–4], revealing chronological information of ore body formation [5], evaluating the economic potential of ore exploration [6,7], and in the provenancing of archaeological materials. In archaeology, the method of comparing the Pb isotopic “fingerprints” of artifacts and other materials to that of ores has been widely applied despite initial and ongoing debates regarding their application and overall validity [8–11]. Indeed, the approach has had a long and controversial history of proof of concept, setback, and eventual acceptance [12–17]. Often cited in these debates, and the basis for the usefulness of Pb isotopes in provenance studies, lies in the relative abundances of four stable isotopes (^204^Pb, ^206^Pb, ^207^Pb, ^208^Pb), of which ^204^Pb is primordial and has no long-lived radioactive parent. Primordial Pb can therefore be leveraged to model the elapsed time since the initial formation of a given Pb-bearing mineral. The abundance of primordial Pb remains unchanged while the concentrations of the other three increase over time due to the radioactive decay of their U and Th parent isotopes of ^238^U, ^235^U, and ^232^Th, respectively. The relative abundances of these four isotopes impart age information and often a unique geochemical signature in ores [18–24], of which the latter is commonly the focal point in archaeological provenance studies.

Throughout Chinese history, Pb was commonly used in a variety of materials and processes including bronzes [25, 26], lead-barium (Pb-Ba) glasses [27], glazes [28], pigments [29], firearms [30], and in silver production [31]. As a result, Chinese archaeologists and historians have taken interest in Pb, resulting in an increased number of provenance studies with particular focus on the circulation of bronze metalwork dating to the Shang-Zhou period (ca. 1200–250 BC) [32–34]. Despite this increase however, no attempt has been made to create a centralized database of assessed Pb isotope analyses for archaeologically relevant Pb ores and other related metal deposits. Such databases already exist in Europe [35, 36], and North [37] and South America [10], and have been shown to be indispensable in provenance studies. In addition to a collection of Pb isotopic signatures, these databases provide informed geochemical information. Two of the most important applications of such databases are in showing the isotopic variability of the calculated model ages, ^238^U/^204^Pb and ^232^Th/^238^U ratios in ore deposits [38], and in facilitating the identification of isotopic anomalies such as geologically old Pb, Thorium(Th)-rich Pb, Uranium(U)-rich Pb, and highly radiogenic Pb [39, 40]. Highly radiogenic Pb, in particular, has been identified as a unique marker in the provenancing of Chinese bronzes and other non-metal materials [41].

In order to create a database useful for the provenancing of Chinese cultural materials, this paper compiles all currently available Pb isotope data for galena and K-feldspar in China. It then, interprets these data in regard to the geochemical zoning and genesis of Pb in underlying tectonics and metallogenic belts. The database is also used to discuss the fundamentals of Pb isotope geochemistry and its extension to, and limitation in, provenance studies, followed by a review of current scholarship on ore geology concerning Pb isotope characterization. We then outline the large-scale geochemical zoning and regional-scale variability between ore tectonics in China, followed by, and, perhaps most important, three case studies for Warring-States coinage [42], Qin State ritual vessels [43], and Tang glazed pottery [44]. These studies demonstrate how the assembled database, and its geochemical implications, provide new insight into the supply and circulation of Pb in China. These data are plotted in ternary rather than bivariate diagrams. Ternary diagrams are more concise and better at comparing sets of Pb isotopes from several sources.

### Tectonic terranes and lead isotopes in China

Two monumental monographs pioneered the characterization of lithosphere plates in China using Pb isotope analyses from sulphide ores and K-feldspars [45, 46, 47]. These studies primarily divided eastern China into a number of geochemical blocks (North China, South China, the Yangtze, and Northeast China) to investigate associated geodynamic processes of crustal formation. The results show that continental crusts in China are isotopically heterogeneous across different terranes and tend to form distinct geochemical provinces [48]. Along the blocks of these provinces exist Pb isotope steep zones, or, geochemical boundaries, that are separated by geological faults [46]. Among them, the border regions between the North China Craton (NCC) and Hinggan (41°N), and between the NCC and Yangtze Craton (31° N latitude), are quite distinct [48]. Ore deposits forming along these boundaries could potentially exhibit unique isotopic ratios. Given these distinctions it is thus essential to understand the formation of Chinese tectonic terranes that constrain relevant metallogeny with specific geochronological and geochemical Pb isotope signatures.

**Fig 1:**
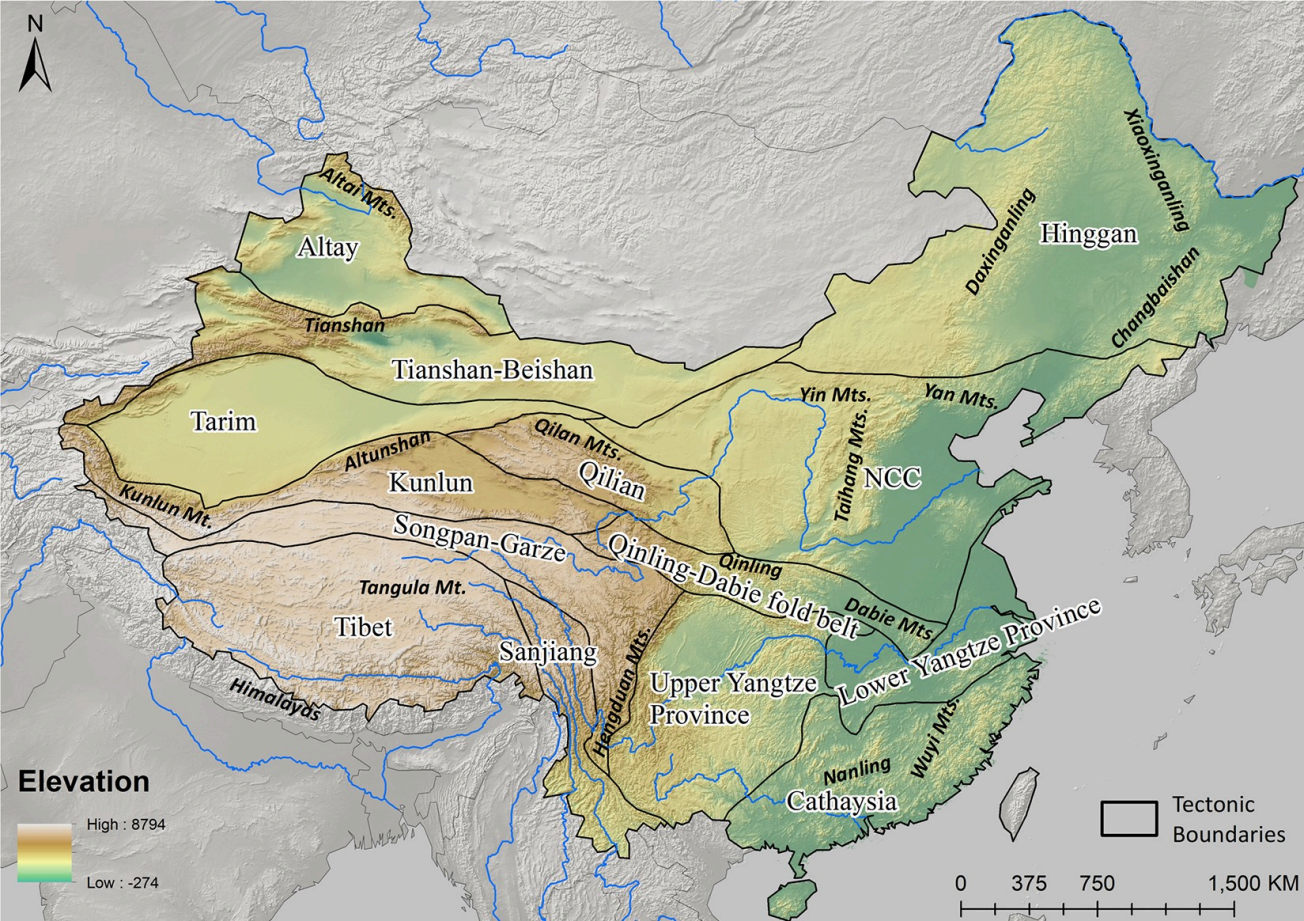
Simplified map of tectonic terrains in China.

**Fig 2:**
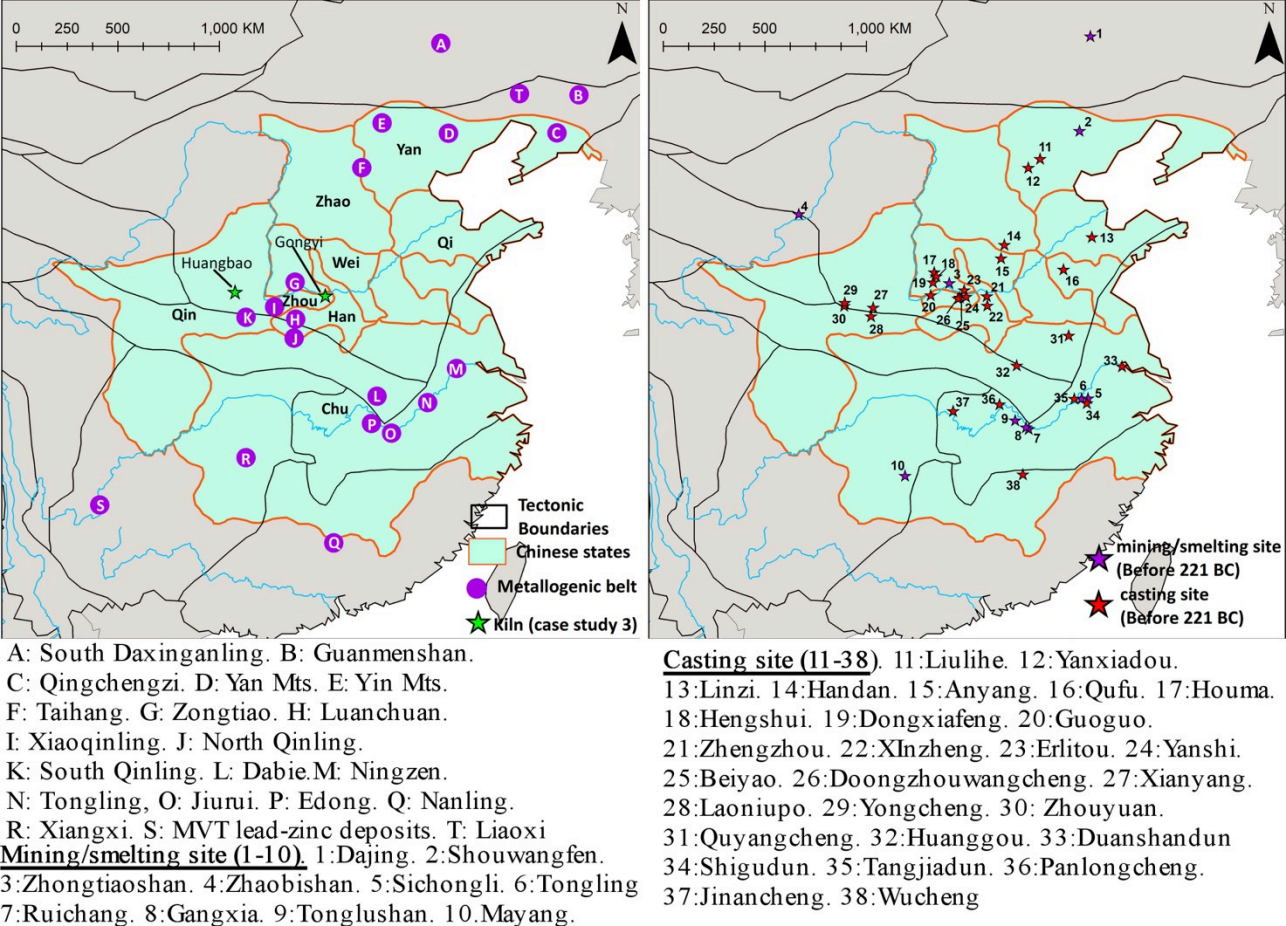
Map of the major metallogenic belts in eastern China. Also shown are the Warring State Period Chinese states (475–221 BC), relevant prehistoric mining/smelting and casting sites, and the locations for sites discussed in the case studies.

The geological and geochemical setting of present-day China is the result of the collision of major tectonic plates in southeastern and eastern Asia, resulting in several Precambrian cratons and Phanerozoic orogenic belts (Fig 1) [49–60]. The primary tectonic provinces include the NCC, Tarim Craton, and South China Block (SCB), which is divided into the Yangtze Craton and Cathaysia. Along the boundaries of these continental plates lie a series of orogenic zones, namely: Altay, Tianshan-Beishan, and Hinggan (forming part of the Central Asian Orogenic Belt); the Qilian/Qinling-Dabie fold belt (also known as the Central China Orogen); the Kulun/Songpan-Garze Orogen; and the Tibet/Sanjiang fold belt (Himalayas). In regard to the archaeological significance of these provinces, mining/smelting activities have mainly been identified in the eastern part of China in the Middle-Lower Yangtze River Valley (Edong, Jiurui, and Tongling), Taihang, Zhongtiaoshan, South Daxinganling, South Qinling, and Nanling (Fig 2) [61, 62]. It is also important to note that the majority of non-ferrous metal resources in China are hosted in Mesozoic mineralizations [63], and approximately 46.5% of Pb-Zn deposits in China were formed during the Yanshanian period (208–90 Ma) [64]. If these deposits happen to have had similar geological histories then their resulting Pb isotopic ratios could be, irrespective of the tectonic terranes that they reside, indistinguishable.

The NCC is the oldest continental block in China and is comprised of Archaean to Paleoproterozoic (~3.0–1.8 Ga) basement rocks that were extensively worked and deformed by repeated magmatic activity [60], which led to variability in ore-forming materials and subsequent Pb isotopic ratios. The central part of the NCC is represented by a north-trending 1,200 km long and 100–300 km wide Palaeoproterozoic suture zone that comprises a series of metallogenic belts along mountainous ranges. The southern edge of this region hosts the Tongkuangyu porphyry Cu deposit in the Zhongtiaoshan region [65], which was a vital source of Cu in the Central Plains of China during the Bronze Age (ca. 1500–250 BC) [61]. The Yangtze Craton, divided into the Cathaysia and the Upper and Lower Yangtze provinces, on the other hand, is mostly comprised of younger Neoproterozoic basement and two terranes that were amalgamated to form the SCB ~800–760 Ma [59]. Within the Lower Yangtze province is one of the most important metallogenic belts in China with more than 200 porphyry, skarn, and stratabound ore bodies of polymetallic ores that are clustered in seven districts along the Middle-Lower Yangtze metallogenic belt [66]. According to recent textual and archaeological evidence, this region was intensely exploited by Chinese prehistoric miners, which is evidenced by hundreds of smelting sites. These sites may have supplied raw metal for the manufacture of Shang-Zhou period bronzes in the Central Plains [61]. The copper from skarn deposits in Tonglushan (Edong), Ruichang (Jiurui), and Shizishan (Tongling) were especially important for these bronzes [61].

The Qinling-Dabie fold belt formed during the late Paleozoic and Mesozoic convergence of the NCC and the Yangtze Craton, involving multi-stage tectonic processes such as oceanic subduction, terrane accretion, and continental collision [67]. In the east, the Dabie orogenic belt is marked by Triassic ultrahigh-pressure metamorphic rocks formed by continent–continent collision [68]. The ensuing metamorphism likely caused the preferential loss of uranium in aqueous fluids resulting in elevated Th/U ratios [69]. In the west, the Qinling orogenic belt is characterized by Paleozoic tectonic events of arc–continent collision, followed by late Permian to early Triassic continental collision between the NCC and Yangtze Craton [49, 70]. This region is traditionally subdivided into the North and South Qinling belts, which converged during the Devonian period (ca. 359–420 Ma) [71–73]. The late Neoproterozoic and early Paleozoic sedimentary sequences in the South Qinling belt are similar to the strata system within the Yangtze Craton, indicating a close geological affinity between these two tectonic units [74]. The formation of these units is also historically important since records indicate ore exploitation took place in South Qinling during the medieval to pre-modern periods (7^th^ to 19^th^ centuries AD), which is supported by modern-day archaeological field surveys [75].

The Hinggan orogeny, situated in the eastern extension of the Central Asian Orogenic Belt is the result of the closure of the Mongol–Okhotsk ocean between the NCC and the Siberian Platform in the Jurassic to early Cretaceous [57, 60]. The Yanshanian tectono-thermal event reworked the juvenile oceanic crust, which resulted in felsic magmatism and the formation of the porphyry, epithermal precious, and base metal deposits in the Hinggan province. The southern slope of Daxinganling is particularly renowned for its polymetallic mineralization of Cu, Pb, Zn, Ag, and Sn [76]. Some of these deposits were exploited by pastoral communities from Northeast China during the end of the second millennium BC (ca. 1100–700 BC), especially the Dajing Sn-polymetallic deposit mined by the Upper Xiajiadian communities [77].

### Lead isotope database

Recent geochemical studies have yielded thousands of Pb isotope measurements from Chinese ore deposits over huge geographical areas, yet this wealth of data has yet to be fully utilized for the provenancing of cultural materials. While these modern ore data may not fully represent past exploited ores, which could have been exhausted or come from large economic deposits that were inaccessible in antiquity, they nevertheless provide a good point of reference for provenance studies and should not be overlooked. Various examples of Pb isotope ratios of metallurgical remains in China correspond to modern ores [77, 78, 79], giving credibility to the use of present-day isotope data. For the purposes of characterizing and provenancing Pb sources, the database mainly comprises galena and K-feldspar samples from after the year 2000. The majority of the Pb isotope measurements prior to this year suffer from high experimental error (~0.5%) and are thus unsuitable for our purposes [80]. A thorough investigation and commentary on the quality and reproducibility of these data for several ore types is given in the S1 Appendix. Lead-rich minerals such as galena, cerussite, and anglesite, due to their high Pb in respect to U and Th, are the best candidates to approximate the relative amounts of each that decayed in current ratios of radiogenic Pb, except in mixed reservoirs [81]. Likewise, K-feldspars from granitic rocks, having a much higher abundance of Pb derived from U, have been used to source metals [82]; however, such sourcing is limited because Pb from hydrothermal fluids may not be geologically associated with ore bodies if they formed outside of granitic plutons [36]. We therefore only use the isotopic composition of K-feldspars to differentiate tectonic terranes on a large scale. In contrast to Pb-rich minerals, the isotopic signatures of Pb-poor ones, including whole rocks and non-Pb ores, can be altered in situ by the radioactive decay of U and Th in associated ores. These altered minerals are unsuitable for most provenance studies unless prior examination ensures that all of the sulfide minerals originated from the same hydrothermal fluid during ore formation.

The database itself is a compilation of all previously published data on galena and K-feldspar samples comprising 5,609 Pb isotope analyses drawn from galena (n = 4057) and K-feldspar (n = 1552). Among these data, however, only 2,615 are of practical use for provenancing since the remainder were made with low precision instruments and unreliable protocols. An additional 90 analyses of sulfide minerals (pyrite, pyrrhotite, chalcopyrite, and sphalerite) are also included in the database for the Middle-Lower Yangtze metallogenic belt due to a lack of sufficient galena ore measurements for the region. This metallogenic belt was an important source of Cu and Pb throughout historic and prehistoric time periods. The Pb isotope data in the database were gathered from journal articles and theses available through the China National Knowledge Infrastructure (CNKI) website using the following search terms in both English and Chinese: lead isotope (铅同位素), geochemistry (同位素地球化学), and ore-forming material (成矿物质). Data from English language publications were primarily obtained from *Ore Geology Reviews* and *Economic Geology*. Once found, all data were input into an excel document in English and Chinese with corresponding sample location, tectonic and metallogenic unit, fundamental parameters (model age, U/Pb and Th/U ratios), and references. Also included in the database are the conversion variables used to plot the ternary diagrams in this paper. The database has been made freely available at the Harvard Dataverse (https://doi.org/10.7910/DVN/VID3WR). More detail about it can be found in the S2 Appendix.

### Presentation of Pb isotopes

The first ternary Pb isotope diagrams were published by Cannon *et al*. (1961) as a solution to contemporary differences in interlaboratory analytical precision and the inability to accurately measure the low relative abundance of ^204^Pb [83, 84]. Following their plotting methodology, but with reviewed and reliable analyses, and for the purposes of conciseness and simplified comparability of isotopic data, we have also used ternary diagrams throughout this paper. In order to transpose Pb isotopic ratios, that are traditionally presented in bivariate plots, to a ternary system, the data are normalized to 1 by virtue of the shared ^204^Pb denominator after the method given by *Cannon et al*. (1961). We also provide corresponding traditional bivariate plots of these data for comparative purposes in the S3 Appendix. Further detail about the benefits of using ternary Pb isotope plots in provenance studies is forthcoming (Sabatini and Hsu forthcoming) [85]. In addition, an important aspect of presenting and interpreting Pb isotopes is in the statistical treatment of data. Lead isotopes from ore deposits may not comply with Gaussian distributions [86] and therefore non-parametric methods such as kernel density estimations (KDEs) are preferable to visualize isotopic fields [87]. The included case studies will employ these presentation methods; namely, ternary diagrams, fundamental parameters, and KDE estimations.

There is also an alternative way to display Pb isotopes by converting the ratios to fundamental parameters, namely, the model ages ^238^U/^204^Pb and ^232^Th/^238^U, using a two-stage Pb evolution model [38, 88]. This method should also be considered as it offers two advantages: first, it allows one to easily obtain age information about geological sources or tectonic events from which archaeological material derived [38]; and, second, zones defined by U/Pb are potentially robust enough to allow for discrimination between isotopic segments within metallogenic provinces [89].

### The distribution and patterning of Pb isotopes in eastern china

**Fig 3:**
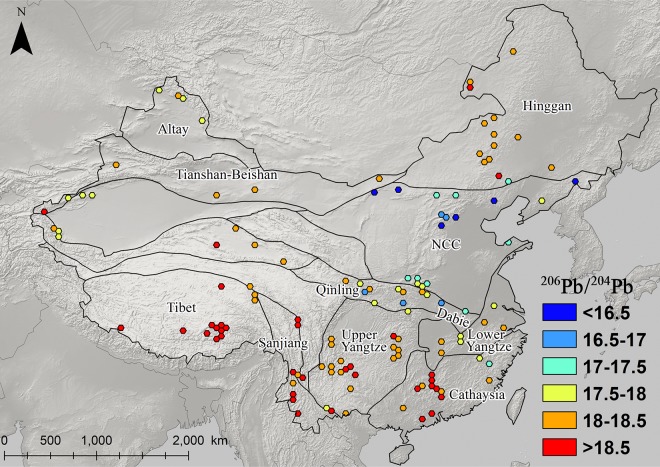
Map of ^206^Pb/^204^Pb ratios for galena ores (n = 2,104). Each color represents the median Pb isotope ratios in a 60 x 60 km hexagonal grid containing at least five measurements.

**Fig 4:**
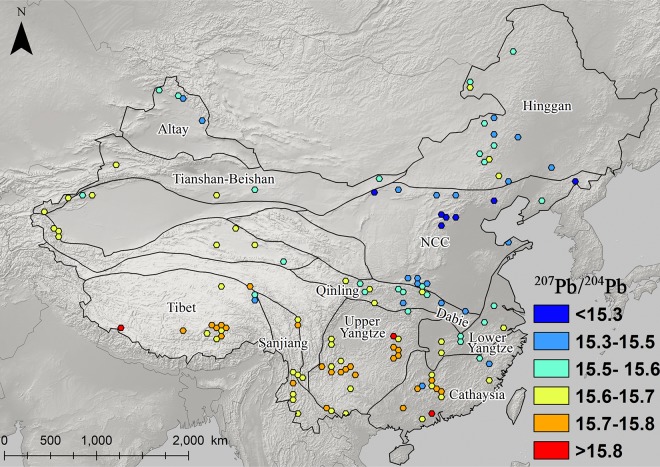
Map of ^207^Pb/^204^Pb ratios for galena ores (n = 2,104).

**Fig 5:**
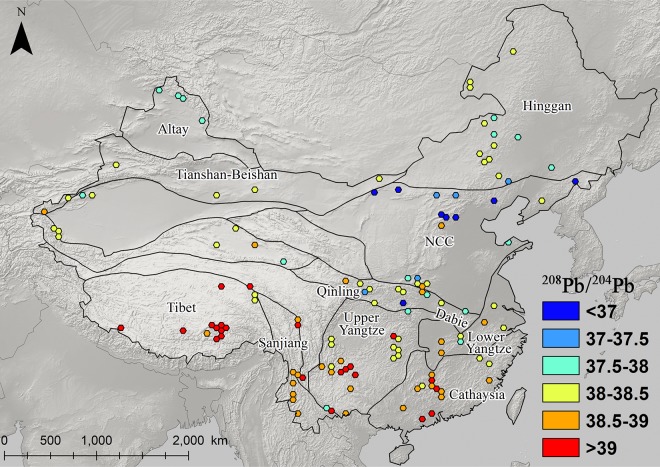
Map of ^208^Pb/^204^Pb ratios for galena ores (n = 2,104).

**Fig 6:**
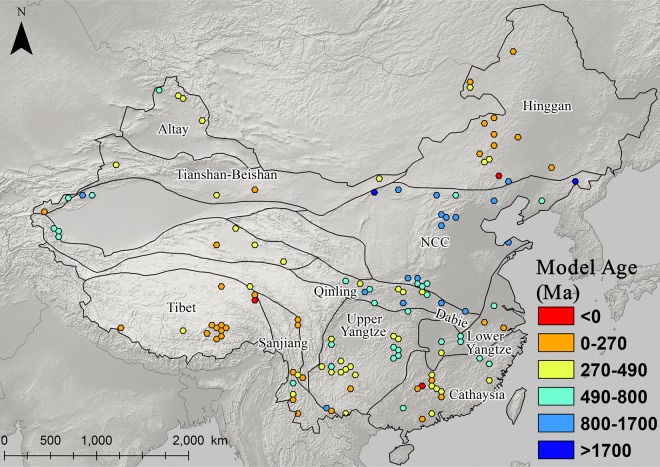
Map of ^238^U/^204^Pb ratios for galena ores (n = 2,104).

**Fig 7:**
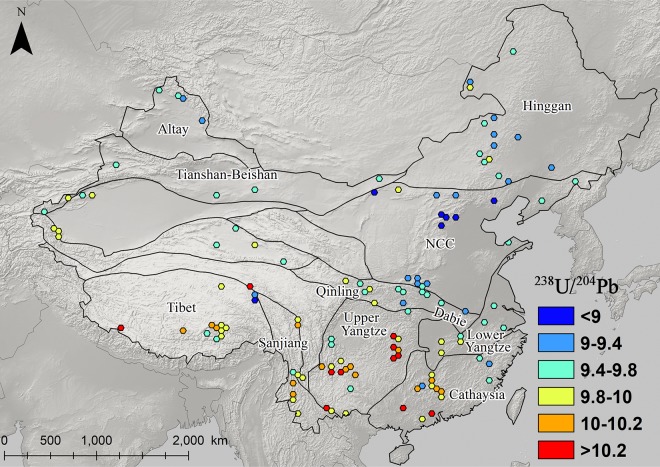
Map of model ages for galena ores (n = 2,104).

**Fig 8:**
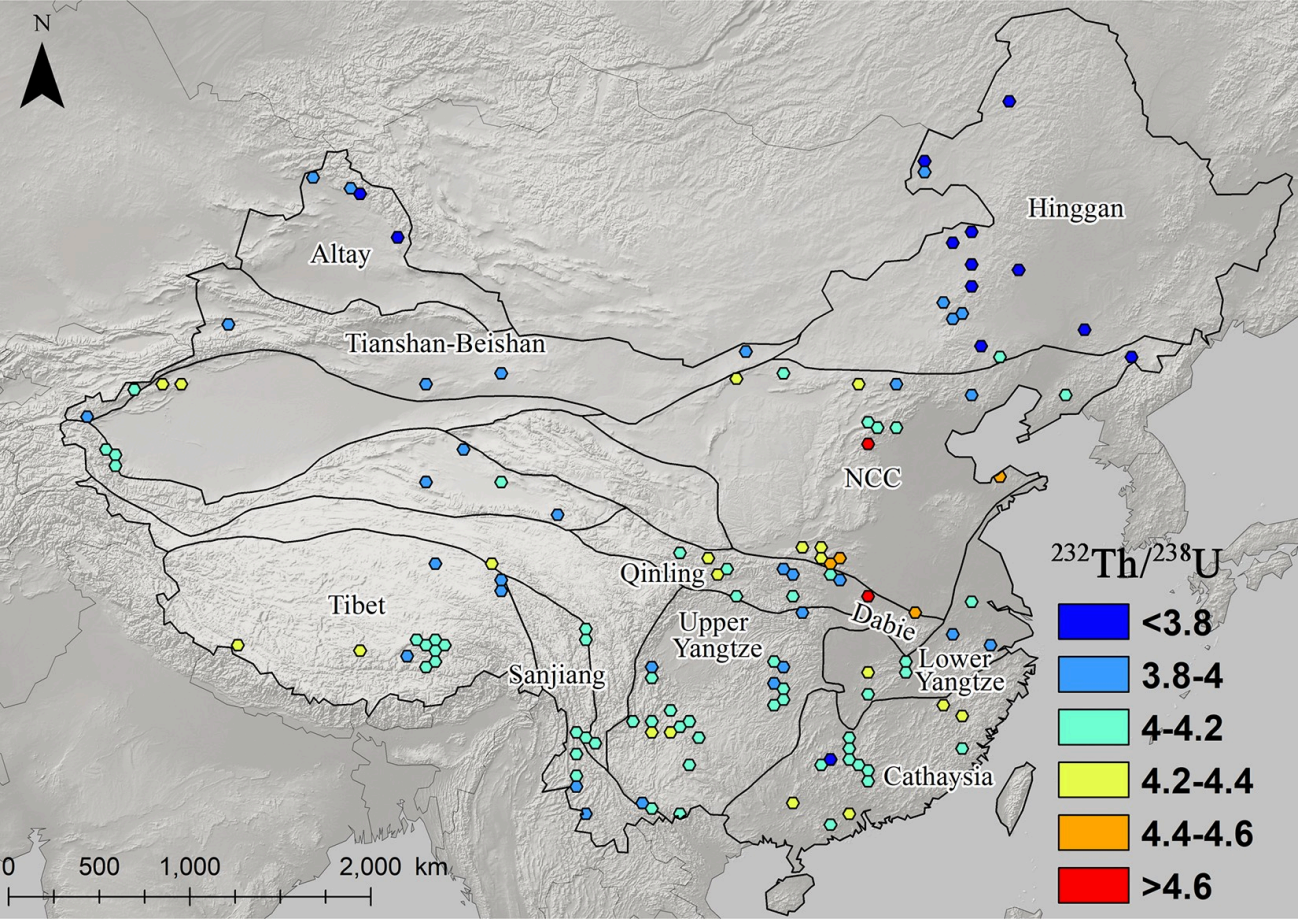
Map of ^232^Th/^238^U ratios for galena ores (n = 2,104).

**Fig 9:**
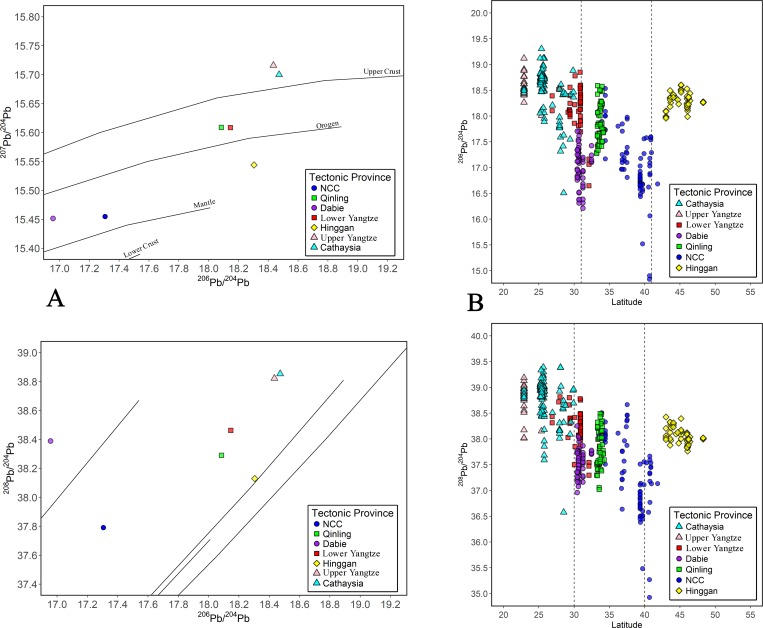
The general isotopic patterning of metallogenic provinces in eastern China. (A) Bivariate plots of Pb isotope medians in individual provinces against plumbotectonic growth curves. (B) ^206^Pb/^204^Pb and ^208^Pb/^204^Pb ratios in K-feldspar (n = 511) by latitude. Two datasets published before 2000 are included due to the higher than usual precision of their analyses [91, 92].

Figs 3–8 show the spatial distribution of three isotopic ratios of galena ores and their inferred parameters, which were calculated from a two-stage evolution model [38]. Their distribution patterns are heterogeneous and discriminate different metallogenic provinces [82]. One of the most recognizable patterns in these ratios are the relatively low radiogenic Pb in the NCC, particularly those ore fields along the geochemical boundary between the NCC and Hinggan province. The medians of these unelevated isotopic values are typically less than 17.5, 15.5, and 37.5 for ^206^Pb/^204^Pb, ^207^/^204^Pb, and ^208^/^204^Pb, respectively (Figs 3–5). From a geochemical standpoint, these values indicate that the tectonic sources of Pb along this boundary are outlined by a low U/Pb ratio (< 9.4), which are similar to those in the plumbotectonic mantle model (Figs 6 and 9A) [90]. The suture zone in the central NCC has a comparatively smaller U/Pb ratio smaller at < 9, suggesting some contamination from lower crust materials. The model ages of ore samples in the NCC are typically older than 800 Ma, which are consistent with local Precambrian basement rocks (Fig 7). Depleted U/Pb and old modeled ages are unique to the NCC, especially along its northern border. The unradiogenic isotopes in the NCC is in stark contrast to the radiogenic ones in the SCB, which has values that are typically higher than 18, 15.6, and 38.5 for U- and Th-derived isotopes (Figs 3–5). An elevated U/Pb ratio (> 10) in the SCB suggests the incorporation of source materials from the upper crust (Fig 9A).

The Qinling-Dabie fold belt and the Lower Yangtze province have intermediate Pb isotope values that fall between the NCC and SCB, but their overall medians are varied (Figs 3–5); galena specimens usually range from 9.4 to 9.8, which is close to the average crustal composition (9.74) defined by the Stacey and Kramer model [86] (Fig 6). The average growth curve of Pb isotopes in their model is comparable to the plumbotectonic model of the orogenic reservoir where materials from the mantle and continental crust mixed to generate magmas forming a convergent boundary (Fig 9A). This reflects the character of the Qinling belt and Lower Yangtze province as being a mixture between the mantle or lower crust-driven NCC and the SCB. In contrast, the Dabie orogenic belt is characterized by low ^206^Pb/^204^Pb and ^207^Pb/^204^Pb, but normal ^208^Pb/^204^Pb (Figs 3–5). This isotopic pattern is attributed to the loss of U relative to Th during local high-grade metamorphism from the collision between the NCC and Yangtze Craton. Therefore, the Dabie Pb isotopic medians are close to the growth curve of the lower crust with low U/Pb and high Th/U ratios (Fig 9A).

To the north, the Hinggan province is also marked by intermediate Pb isotope values similar to those from the Qinling and Lower Yangtze province; however, closer examination shows distinct model ages and Th/U values (Figs 7 and 8). The sources of Pb in the Hinggan formed from relatively younger basement rocks (< 270 Ma) in a Th-depleted environment. This province is isotopically comparable to northern Xinjiang (Altay), and together are incorporated into the larger Central Asian Orogenic Belt [45]. Most Chinese metallogenic provinces, except for the Hinggan, have Th-derived Pb that is above their designated theoretical reservoirs (Fig 9A). Thorium-rich environments in China can be attributed to three factors: first, the lithosphere originally contained Th-rich reservoirs; second, fractionation of U and Th in the crust was caused by intense reworking and melting; and, third, the crustal materials of the lithosphere had already incorporated old Pb from the Archaean age when the overall Th/U ratio (~ 4.65) was greater than the modern continental crust [93]. In addition to galena ores, K-feldspars also exemplify the shift in Pb isotopic ratios over continental crusts in eastern China [47]. Three main isotopic zones can be identified from the south to north; the SCB, NCC, and the Hinggan (Fig 9B). Granitic rocks in the SCB are rich in radiogenic Pb, which declines in the NCC and rises again in the Hinggan. Between these terrestrial plates are transitional zones, also known as geochemical boundaries, represented by the Dabie orogenic belt from ~ 31° N latitude in the south to the edge of the NCC at ~ 41° in the north. They are marked by sharp variations in isotopic values, especially where there is depleted U-derived Pb. The ultrahigh-pressure metamorphism in the Dabie tends to leach out U in rocks, causing low ^206^Pb/^204^Pb but an expected range for ^208^Pb/^204^Pb. On the other hand, the NCC, in addition to local metamorphic processes, underwent intensive volcanic activity in the Mesozoic that contributed to a wide range of Pb isotopic ratios, owing to the mixture of different U-Th-Pb reservoirs. Overall, several distinct isotopic provinces can be inferred in eastern China from Pb isotopes of galena and feldspar specimens, confirming the results of previous research [83, 84]. The unique distribution of Pb isotopes provides a good foundation for sourcing exploited ores. Similar geochemical zoning and boundaries have been broadly established in other continents for the same purpose [94–96].

### The complexities of Pb isotope distribution

While the isotopic characteristics of individual provinces can be broadly defined, subtle differences in Pb isotope ratios sometimes exist in overlapping fields. The homogeneity and heterogeneity across and within these fields can either be an advantage or disadvantage for provenance studies, depending on the genesis of Pb from source materials and the disparity and distance between ores. As the continental crust in China underwent reworking in various amalgamation events, much of the original U-Th-Pb reservoirs were altered through partial melting and deformation. Large scale geologic events such as the Yanshanian cycle could have produced ore deposits with similar Pb isotopic ratios across different tectonic terranes. Meanwhile, smaller regional hydrothermal systems of ore bodies could have rendered heterogeneous Pb isotope compositions as fluids that may have travelled across different source regions and mixed at a deposition site [18]. It is thus essential to explore the degree of overlap and variation of Pb isotopic data to understand the limitations of archaeological provenance research.

**Fig 10:**
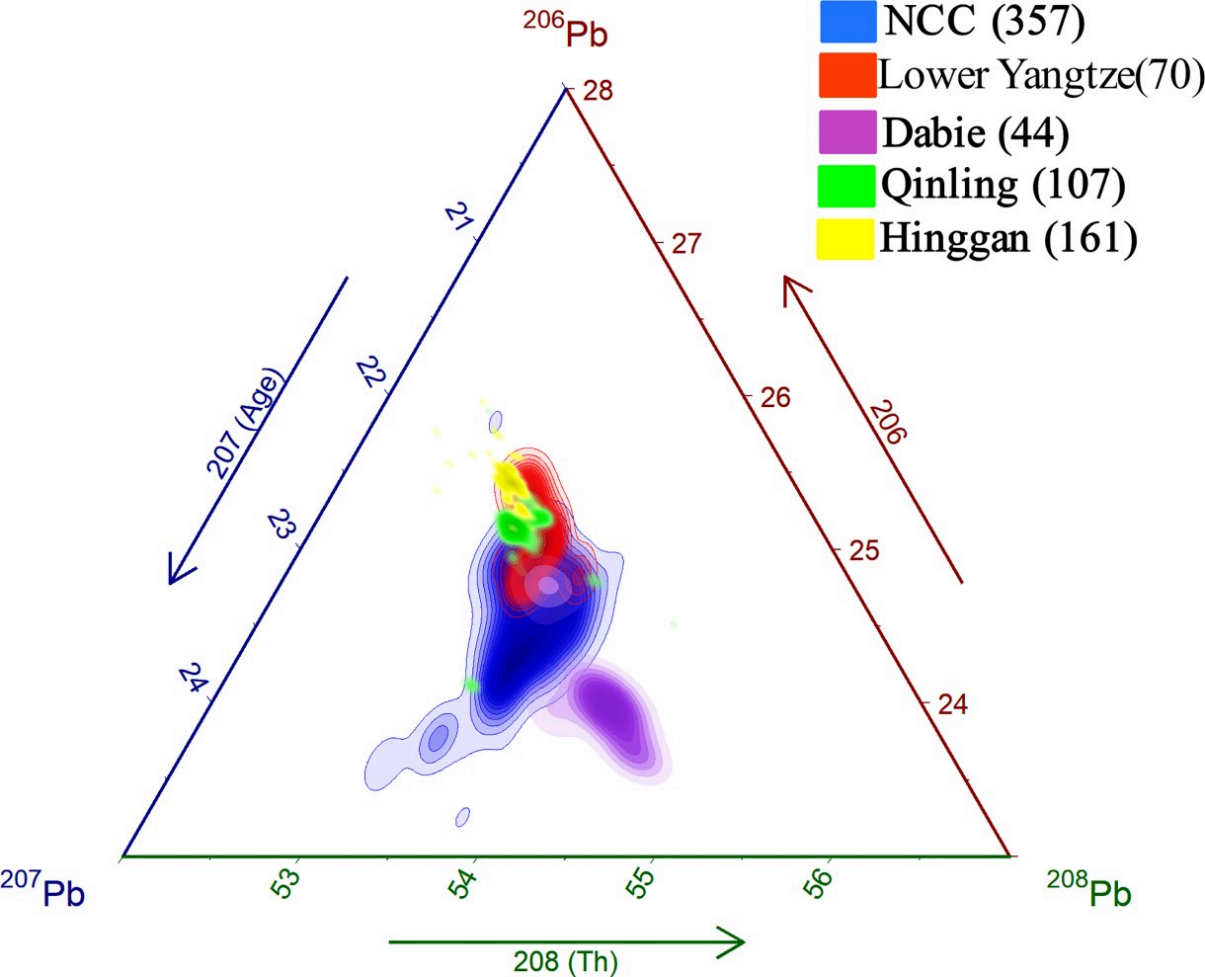
Lead isotope characterization of galena ores from the North China Craton (NCC), Lower Yangtze province, Qinling, Dabie, and Hinggan. The diagram was drawn using the ggtern package in R (http://www.ggtern.com/). The bandwidth for two-dimensional kernel density estimation was selected based on Silverman's “rule of thumb” [99].

Ternary diagrams that summarize the isotopic extent of the NCC, Lower Yangtze Qinling, Dabie, and Hinggan provinces, based on the relative abundances of ^206^Pb, ^207^Pb and ^208^Pb, are shown in Fig 10. The NCC is characterized by a wide variety of ratios, and, in its northern part, the Archaean-Paleoproterozoic cratonic block is overlain heavily by several micro-continent provinces. The enrichment of ^207^Pb in the NCC is an indicator of a source region where radiogenic Pb generated in much older basement rocks. These old rocks were relatively rich in ^207^Pb due to an abundance of ^235^U in the early history of their evolution.

**Fig 11:**
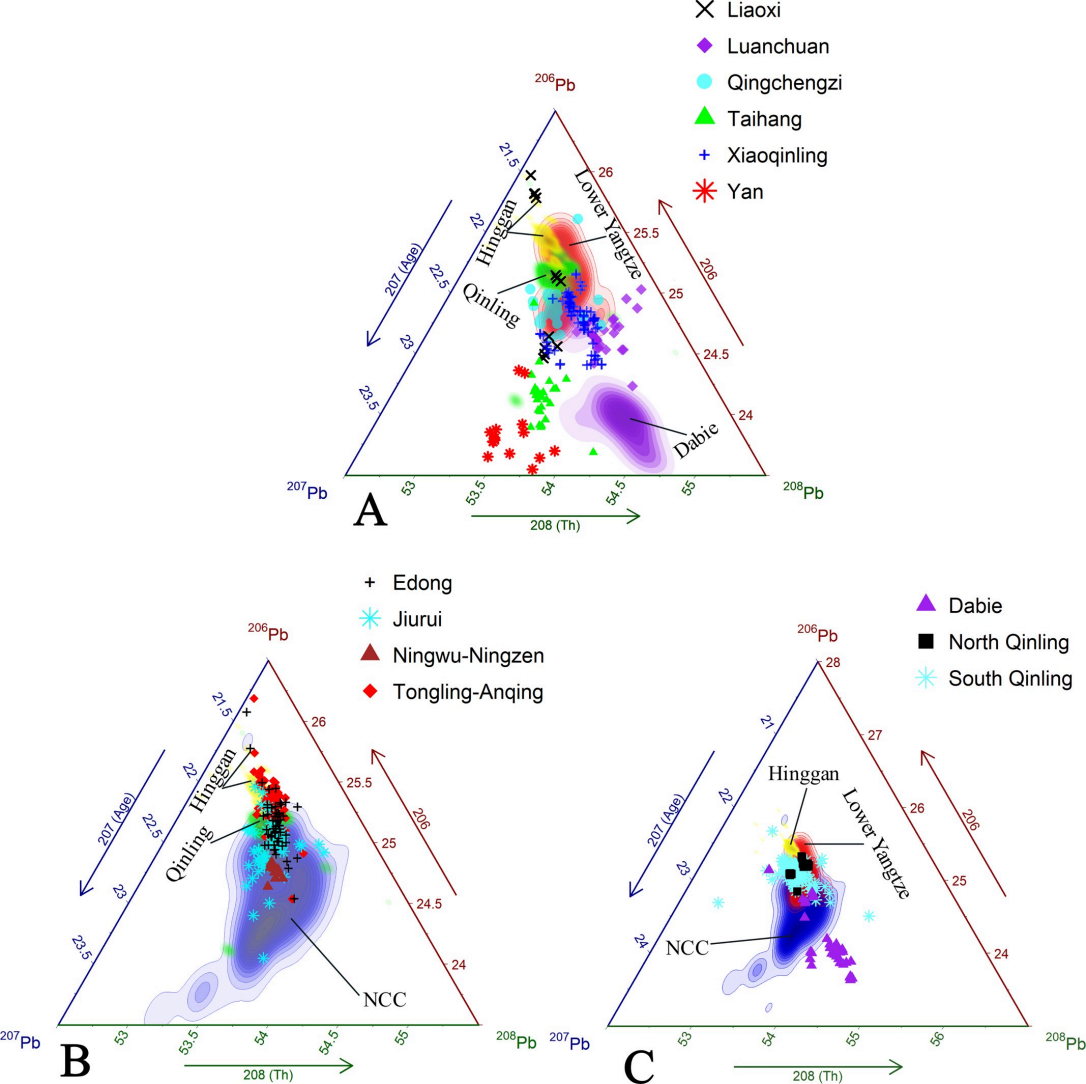
Lead isotope characterization of galena ores from major metallogenic belts in each province. (A) Ternary diagram showing the isotopic variability of metallogenic belts in the North China Craton (NCC). (B) Ternary diagram showing the isotopic variability of metallogenic belts in the Lower Yangtze province. Note that sulfide minerals (n = 90) from the Middle-Lower Yangtze metallogenic belt are also shown. (C) Ternary diagram showing the isotopic variability of metallogenic belts in the Qinling-Dabie.

The NCC contains several metallogenic belts and fields that record the multi-staged development and reworking of Pb from old to young basements (Fig 11A). The Yan mountains metallogenic belt is marked by a rich abundance of ^207^Pb and lack of ^206^Pb. The radiogenic Pb in this region evolved in a much older geological setting than other ore sources in the NCC, with the older rocks tending to have more ^235^U than ^238^U. Taihang is the next aged ore field, and is distinguishable from the precambrian Yan mountain belt by its lesser relative amount of ^207^Pb. Galena ores with relatively high ^207^Pb, such as those in the Yan mountains and Taihang, have thus far been identified along the northern margins of the NCC. Another famous example is the Guanmenshan Pb-Zn deposit in Northeast China. High ^207^Pb in these regions is the result of the recycling of old Pb in the lower crust during the Yanshanian magmatism [97, 98]. Three other ore fields in the NCC, namely Qingchengzi, Xiaoqinling, and Luanchuan partially overlap and are superimposed by the Qinling and Lower Yangtze assemblages, though the Qingchengzi district is more isotopically distinct than the Xiaoqinling and Luanchuan. Liaoxi comprises several multi-stage galena ores with varied ^207^Pb, resulting in varied modeled ages. Similar to the NCC, the Lower Yangtze province also has variable Pb isotopic ratios, but to a lesser degree (Fig 11B). The ore fields in the Middle-Lower Yangtze province, for instance, can be isotopically divided into four zones: Ningzen, Tonling, Jiurui, and Edong. The Ningzen field is characterized by high ^207^Pb and ^208^Pb, corresponding with ^235^U and ^232^Th, respectively. This means that Pb sources from this region are typified by an old modeled age and Th-rich substance. The Tongling, Jiurui, and Edong fields share considerable isotopic overlap that is broadly distributed in the NCC, Qinling, and Hinggan; nevertheless, some unique characteristics can be discerned. For instance, a fraction of sulfide samples from Tonling are ^207^Pb-depleted, indicating younger modeled ages than other ore districts. In contrast, Jiurui contains a number of specimens with relatively old estimated ages.

**Fig 12:**
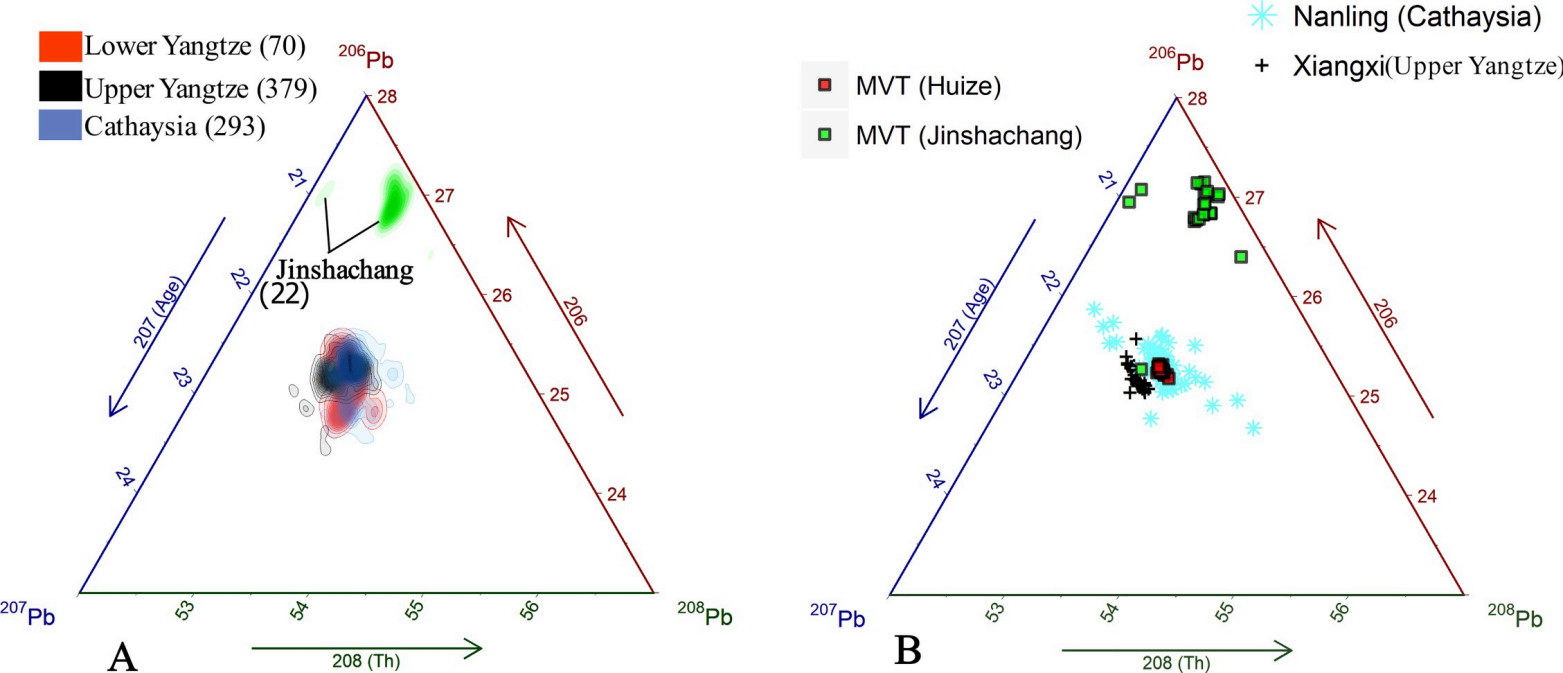
Lead isotope characterization of galena ores from the South China Block (SCB) and major metallogenic belts within. (A) Ternary diagram showing the isotopic fields of the Lower and Upper Yangtze province, Cathaysia, and Jinshachang Pb-Zn deposit. Jinshachang belongs to the Mississippi Valley Type (MVT) ore cluster in Northeast Yunnan. (B) Ternary diagram showing the isotopic variability of the Mississippi Valley Type (MVT) (Huizhe and Jinshachang deposits), Xiangxi, and Nanling metallogenic belts.

The Qinling-Dabie fold belt is comprised of the Dabie and the South and North Qinling (Fig 12C). The central area of the Dabie is separated from other isotopic provinces and is uniquely Th-rich with high ^208^Pb, which formed due to the preferential fractionation of oxidized U to Th during high-grade metamorphism [100]. The Pb isotopic character of the South and North Qinling, in contrast, have some internal heterogeneity, but are otherwise indistinguishable from the NCC and Lower Yangtze province. In comparison the distribution of Pb isotope ratios from the Hinggan province suggests that there was a deficiency of Th (low ^208^Pb) in ore-forming substances (Fig 11). The narrowly plotted range of the Hinggan is likely due to huge hydrothermal fluids that homogenized its overall composition during ore precipitation. The hydrothermal deposits on the southern slope of Daxinganling are connected genetically to Mesozoic dated magmatism [101].

Finally, the metallogenic belts of the Upper Yangtze province and Cathaysia show considerable overlap in the upper part of the Lower Yangtze province (Fig 12A). The Upper Yangtze province is composed of a bimodal distribution of young (high ^207^Pb) and old (low ^207^Pb) modeled aged Pb with the Upper Yangtze province overlain by the Cathaysia. The young-aged galena ores from the Upper Yangtze province derive mostly from Mississippi Valley Type (MVT) carbonate-hosted Pb-Zn deposits. The mineralization features a multi-stage reworking of Pb with varied amounts of radiogenic Pb from sedimentary rocks that accumulated during the ore-forming process [102]. The main body of the metallogenic belt is located in Northeast Yunnan and comprises several deposits, such as the large Pb-Zn one at Huize, with Pb isotopic signatures that have a restricted range (Fig 12B) [103]. However, these MVT ores also host highly radiogenic Pb-Zn deposits similar to those found in Jinshachang, giving a high U/Pb ratio and futurist modeled age [104]. This deposit is considered one of the most likely sources of highly radiogenic Pb for late-Shang period bronzes [34]. Another important Pb-Zn mineralization to consider is the one in Xiangxi, which contains more abundant ^207^Pb than is typically found in MVT. Nanling, on the other hand, displays a similar isotopic signature as the primary MVT galena ores and can be easily differentiated from Xiangxi orebodies.

### Case studies

There are three important attributes of Pb isotopes that make them effective in provenance studies: first, unique and identifiable isotopic variations can occur in ore deposits due to the preferential fractionation of the U-Th-Pb system in hydrothermal processes within continental crusts [105]; second, Pb isotopes are immune to fractionation by redox (weathering) reactions of primary sulfide ores thus retaining their tectonic basement signatures [6]; and, third, Pb isotopes are only marginally, yet unobtrusively so, altered by anthropogenic pyrometallurgical processes [106–108]. In the first instance, fractionation processes, coupled with the time-controlled radioactive decay of parent isotopes, differentiate source substances from different thermo-tectonic settings. In the second, the weathering of complicated continental crust can result in significant variation and anomalies in subduction zones in accretionary wedge and melange formations, such as those in the Yanshanian subduction and Qinling mountains, between geochemical boundaries of tectonic plates. For instance, the Mujiazhuang copper (Cu) deposit of the Zhashan polymetallic district in Qinling resides in a fault that contains ore minerals with highly radiogenic Pb (^206^Pb/^204^Pb ~22.14, ^207^Pb/^204^Pb ~15.85, ^208^Pb/^204^Pb ~40.97) [109]. These ores are in stark contrast to other epigenetic mineralizations with common Pb in the same metallogenic district. And in the third, review of the degree of fractionation of lighter ^204^Pb has been shown to be relatively inconsequential in provenance studies [108].

Together the above attributes of Pb isotopes suggest that they are ideal for provenancing, however their usefulness to archaeologists is not without several caveats. Lead isotopic signatures are often not unique between orebodies or have considerable overlap across ore fields [16], can be heterogeneous within single ore deposits and minerals [110], and may have been dramatically modified by both geological and anthropogenic mixing processes [111, 112]. For instance, the Alpine orogenic event during the Late Mesozoic period resulted in Pb-Zn mineralizations in Southeast Iberia, Tunisia, the Balkans, and Anatolia with similar Pb isotopic signatures [36]. Lead within a single ore deposit may have also had successive stages of mineralization, through the remobilization of preceding Pb-bearing minerals, and can either retain the isotopic composition from earlier paragenesis or have a varied ultimate isotopic pattern [113]. The degree of isotopic modification in such cases lies in the Pb content of the hydrothermal fluid present during each phase. Finally, the most challenging issue for Pb isotope provenancing lies in the mixing and recycling of raw and finished materials resulting in archaeological objects that no longer bear the signature of a single mine or orebody. The above complications are considered in the three case studies that follow.

**Fig 13:**
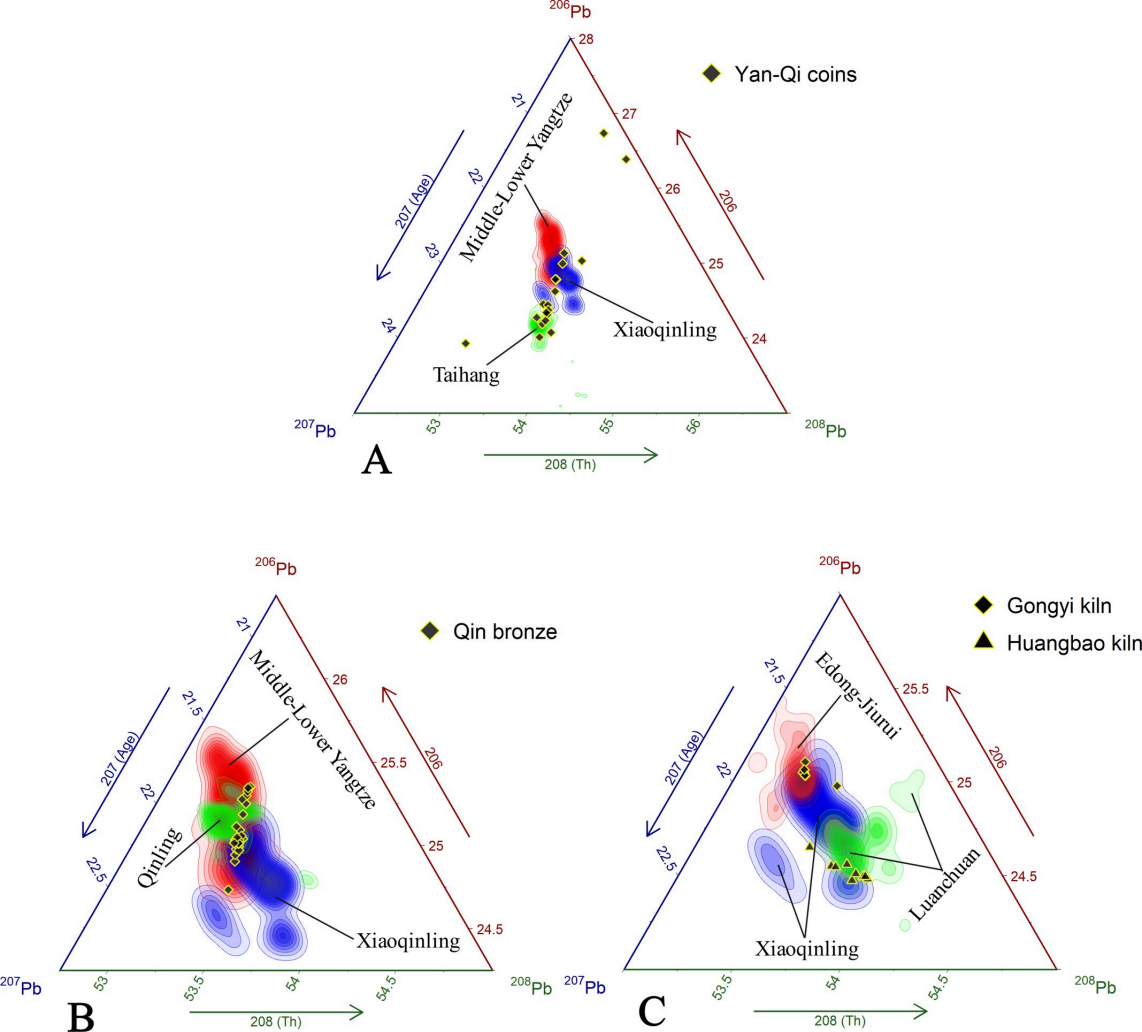
Application of the Pb isotope database in three archaeological provenance case studies. (A) Case study of Yan Qin coins and potential Pb sources. (B) Case study of Qin State bronzes and overlapping isotopic fields. (C) Case study of Tang Sancai Pb glaze and its potential sources.

Our first case study revisits the sourcing of copper-based alloy coinage from the Warring States period (Fig 3). A past study showed a regional difference between coins issued by the Yan and Qi States with some containing distinctly low Pb isotopic ratios, which were interpreted as having derived from sources in the Guanmenshan and Qingchengzi ore belts [42]. However, a broad comparison of these data with the compiled ore database indicate that the Pb, except for two coins that contain high ^206^Pb, likely originated from sources in the NCC and the Middle-Lower Yangtze belt (Fig 13A). Coins with relatively high ^207^Pb match the Taihang metallogenic belt, suggesting the Pb is from geologically old crustal material. This finding is important because it suggests that ore exploitation of the Taihang began earlier than the presupposed Medieval period. Some of the coin data also overlaps with the Xiaoqinling and Middle-Lower Yangtze ore belts, and thus cannot be associated with either. Their isotopic signatures may indicate that they contain a single or combination of Pb from these sources and are the result of anthropogenic mixing.

Despite there being no archaeological evidence supporting the mining of ores in the Taihang and Xiaoqinling belts during the Warring States Period, the ores are, nonetheless, geographically close to the Yan, Wei, and Han States thus warranting their consideration. The Middle-Lower Yangtze belt, on the other hand, is well known to have been a source for metals under the Chu State in southern China [114]. In all, the coin data is well represented by the database. The dispersion of coins shown in this case study, that contain Pb from sources outside of the state they were minted, attest to the extensive interaction between states during this time period.

The second case study examines Pb in bronze vessels from the Qin State that dates to the Spring-Autumn period (ca. 776–457 BC, Fig 2). The Pb in these vessels have several possible sources (Fig 13B), however a past study has attributed it solely to ores from the Qinling mountains despite significant overlap of the artifact and source data [43]. The original study’s interpretation is an attractive one given the location of the ore within the Qin State, however it is an oversimplification when the vessel data is more closely scrutinized. Many of the vessels have Pb ratios that plot well outside the range of the Qinling belt with higher ^206^Pb and both higher and lower ^207^Pb. Comparison of these Pb signatures to those in the database in fact place them within the range of the Middle-Lower Yangtze and Xiaoqinling belts. These two possible ore sources should not be dismissed given the significant mismatch of the Qinling with the vessel data despite their significant distance from the vessels themselves. Ore data from the Xiaoqinling belt, also within the Qin State (Fig 2), is particularly important, because it indicates the possible early exploitation of Pb deposits in this area. However, given the dispersion of the vessel data among these two sources it is impossible to identify either as the sole source of Pb in the vessels. Lead isotopic similarities between these geological sources are possibly due to the large-scale Yanshanian magmatic activities that homogenized the isotopes in these different ores. Qin bronzes are therefore best interpreted, for the time being, as having multi-source origins or one due to anthropogenic mixing.

The third, and final case study, examines the origins of Pb in Tang Sancai, a type of glazed pottery manufactured during the Tang Dynasty (618–907 AD) [44]. Sancai (三彩), literally translated as “three colors” were prepared with Fe and Cu in different amounts to achieve green, yellow, and white decorative colors [115]. The glazes, regardless of color, generally contain more than 50% PbO [116], making them well suited for Pb provenancing. Previous studies have identified three kiln sites used in the pottery’s production with each made using clay characterized by a unique chemical pattern, which may indicate preferred or limited access to raw materials [117]. Likewise, glaze materials from two of these kilns (Gongyi in Henan Province and Huangbao in Shaanxi Province, Fig 2) show two distinct Pb isotopic compositional clusters; Gongyi glaze from the Yangtze Craton. and Huangbao from the North China Craton [37]. The provenance of the glazes used in these kilns was left unaddressed in prior studies, however using the assembled Pb isotope database it is relatively simple to see the relationship between the Pb isotopic ratios of the glazes and two particular metallogenic regions. Fig 13C shows that the Sancai glaze from Huangbao was likely supplied by the Xiaoqinling metallogenic belt and adjacent Luanchuan Cu-Mo polymetallic deposits in the NCC, and glazes at the Gongyi kiln by Edong-Jurui Cu polymetallic ore deposits in the Middle-Lower Yangtze belt. Each kiln therefore corresponds well with a single source region affirming the broad conclusions made by previous studies but with additional supportive scientific evidence.

## Conclusion

In order to make informed interpretations of Pb isotopes in provenance studies, one must consider the geochemistry and dynamic geological history of crustal formations that host ore deposits. In China, these deposits can be broadly separated into several metallogenic provinces by their tecontic ages or the metamorphism of basement rocks. The older continental plates, such as the NCC, contain the least radiogenic Pb as opposed to the relatively high radiogenesis that occurred in the younger SCB. High-grade metamorphism in the Dabie lithosphere resulted in low Th/U ratios, which are characteristic of lower crustal materials. Unique Pb isotopes usually occur along the geochemical boundaries between provinces such as the metallogenic belts in the Yan, Taihang, and Dabie mountains. The distinctiveness of these ore deposits may allow one to accurately distinguish Pb sources, however it is important to understand that significant overlap occurred between deposits that formed during the Mesozoic period under similar geological conditions. Examples of these similarities can be found in the Middle-Lower Yangtze, Qinling, and Xiaoqinling belts. As a result, some artifacts cannot be assigned to a specific source unless backed by additional scientific, archaeological, and textual evidence.

The applicability of modern ore data to ancient and historic provenance studies is usually hampered by the fact that past ore mines have not been found, were exhausted due to intense mining in antiquity, or were destroyed by historic and modern mining operations. The database compiled in this paper is a collection of Pb isotope analyses from modern mining sites that are generally applicable and useful for ancient and historic provenance studies, however it is possible that ores exploited in the past were markedly different and featured heterogeneous segments that no longer exist for comparison. The use of Pb isotope data acquired from modern mining sites should therefore be treated as broadly representative of the signatures that existed in the past. In addition, it is essential that archaeometallurgists understand the possible variation in ore mineralizations and endeavor to carry out well-designed sampling strategies to identify potential isotopic variability of multi-phase formations that can be present in a single mine or over a larger source. For instance, most vein-type sulfide deposits in China are in fact polyphasic with paragenesis that may have bore different Pb isotopic ratios. In light of the aforementioned lack of old and representative Pb isotopic ore data, there is an alternative method to retrieving past isotopic signatures by measuring the ratios in recovered metallurgical debris such as inclusions and/or slags [118]. These data, similar to the ratios from the ores themselves, are best applied in provenance studies when coupled with supportive archaeological and/or textual evidence. In all, the best practice in archaeological provenancing is therefore to consider ore geology, mixing, contamination and variability in samples, and, without question, supportive historical and archaeological information. Provenance studies should be an all-inclusive effort rather than a “silver bullet” approach in archaeology. The compilation of our database strengthens the footing of provenance studies overall, however it does not fill the void of comprehensive scholarship.

In conclusion, we believe that the compiled database and case studies discussed in this paper are but another step forward in the progress of providing new insight into artifact provenancing using Pb isotopic data. The intelligent application of the database, and its future expansion, as has been shown instrumental in its European counterpart, should become a fundamental part of any provenance study in China moving forward. Furthermore, the ultimate success of provenance studies and good scholarship in China will undoubtedly require the open accessing of data and the centralization of a Pb isotope database. The case studies in this paper prove that such a database is a powerful tool when applied properly. The success of the chosen case studies also strongly suggests that previously provenanced artifacts should be reevaluated using the database with more recent accompanying supportive evidence.

## Supporting information

S1 Appendix Assessment of data quality. (PDF)

S2 Appendix Contents of the database. (PDF)

S3 Appendix Bivariate diagrams. (PDF)
