# Supplementary material for: A geochemical characterization of lead ores in China: An isotope database for provenancing archaeological materials
Source: PLoS One. 2019 Apr 24;14(4):e0215973. doi: 10.1371/journal.pone.0215973 (PMC6481871; doi:10.1371/journal.pone.0215973)
Supplement: S1 Appendix — (PDF) [file pone.0215973.s001.pdf]

S1 Appendix Assessment of Data Quality

The compilation of published data from different sources raises three major issues; namely, the accuracy of analyses over time, reproducibility of measurements between different laboratories, and the degree to which isotopic ratios represent ore minerals.

(1) Data Accuracy Between Publications

Since the compiled database in this paper contains Pb isotope analyses published between 1979 and 2018, it is crucial to assess the accuracy of the measurements. We initially searched each written source of data for information regarding analytical protocols, laboratories, instruments and experimental errors, and most analyses reported before the year 2000 provide little or no detail. Pre-2000 datasets tend to exhibit wide disparity between isotopic ratios and poor consistency compared to post-2000 ones. This observation is evidenced by analyses of galena samples from Jinding and Huize lead-zinc deposits in Yunnan, Southwest China. There is a huge discrepancy between galena data of Jinding deposit reported before and after 2000 (Figure 1A). The early datasets are characterized by huge isotopic variation as opposed to the much restricted isotopic field of the recent ones. Interpretations based on these early data are therefore highly erroneous, showing sources of Pb from the mantle but in fact the major ore-forming substances should derive from the upper crust in agreement with studies of S, C, H, O, and Sr isotopes [1]. Likewise, galena samples from the Huize deposit demonstrate isotopic variability of early datasets in contrast to the confined range of recent ones. Ore-forming materials in Huize also come from the upper crust [2], and not the mantle as suggested by earlier measurements. As a result, our discussion does not consider data published before 2000, except for a few publications with detailed information about their analytical procedures [3,4,5,6].

(2) Inter-Laboratory Differences

Although a deliberate choice of post-2000 datasets greatly increases the credibility of our interpretations based on published analyses, it is possible that different experimental outcomes of the same material arose from different laboratories. Each lab uses their own particular equipment and methods such as thermal ionization mass spectrometry (TIMS), multi-collector ICP-MS (MC-ICP-MS), and femtosecond laser-ablation multi-collector ICP-MS (fs-LA-MC-ICP-MS). Figure 2 shows the slight inconsistencies of isotopic measurements from different laboratories for the same ore deposits (Jinding and Huize). It can be seen that results from TIMS generally exhibit more variable isotopic compositions than those from MC-ICP-MS and fs-LA-MC-ICP-MS. The fs-LA-MC-ICP-MS method is particularly characterized by *in-situ* Pb isotope analysis that allows for micro-scale measurements of ore minerals formed in different mineralization stages. This is in contrast with the hand-picking analysis of minerals for TIMS and MC-ICP-MS, which may have become contaminated by organic inclusions and other sulfide impurities from multiple stages [7]. However, the overall Pb isotope variations caused by different laboratory methods are fairly small and do not affect the final interpretation as all analyses suggest an upper crust origin for ore-forming in both deposits. In addition, ore minerals in these studies were sampled from different

locations in the orebodies and it is likely that the variation reflects slight heterogeneity within the deposits. We acknowledge the potential analytical differences between different instrumental analyses, however they have little effect on our overall interpretations.

(3) Precision of Different Ore Types

Although the interpretation of Pb isotope data in this paper is primarily based on analyses of galena due to its resistance to isotopic alteration, some archaeologically important ore districts along the Middle-Lower Yangtze metallogenic belt were incomparable because they do not contain enough analyses of galena data to fully characterize ore-forming fluids. As a result we have had to use other sulfide minerals (pyrite, pyrrhotite, and chalcopyrite) as substitutes to approximate likely sources of Pb. This raises a potential issue of whether these Pb-poor minerals can be truly representative of the isotopic signatures of metal fluids since as the accuracy of these measurements depends on the initial U/Pb and Th/Pb ratios, the fluid-rock interaction, and fluid mixture between multiple Pb. In addition, the low Pb concentration in pyritic minerals tend to affect the precision of ^{204}Pb-based plots. Figure 3 shows the comparison between isotopic values of different ore types from the Middle-Lower Yangtze belt. Although Pb-poor minerals in both the Tongling and Juirui districts are relatively more varied than galena samples, they still correctly indicate magmatic sources of lead in agreement with our current understanding of ore fluids of the Middle-Lower Yangtze belt [8,9]. Unfortunately, there are currently no galena analyses available in the Edong district, but both pyrite and chalcopyrite samples point to Pb of magma origin [10].

In sum, it is no doubt preferable to utilize galena for the characterization of ore-forming fluids as its composition is less affected by subsequent in suite growth of radiogenic lead. However, the Middle-Lower Yangtze metallogenic belt is one of the most important metal sources for both prehistoric and historic copper and lead production in China and we believe, we think it is necessary to provide any “potential” isotopic field information for these orebodies while noting their limitations and finite conclusions. To minimize the less precise measurements of Pb-poor minerals, we will present those data in ternary diagrams based on higher precision of ^{206}Pb, ^{207}Pb, and ^{208}Pb.

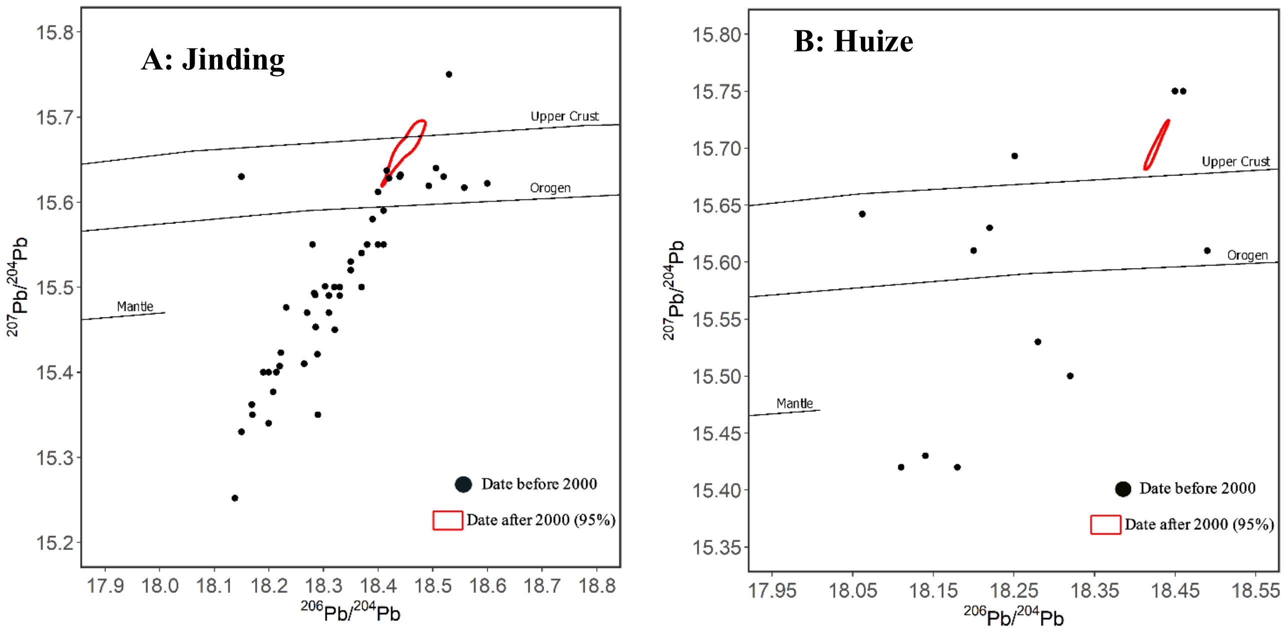

Figure 1. Comparison between isotopic values of galena samples published before and after 2000. A: Jinding. B: Huize.

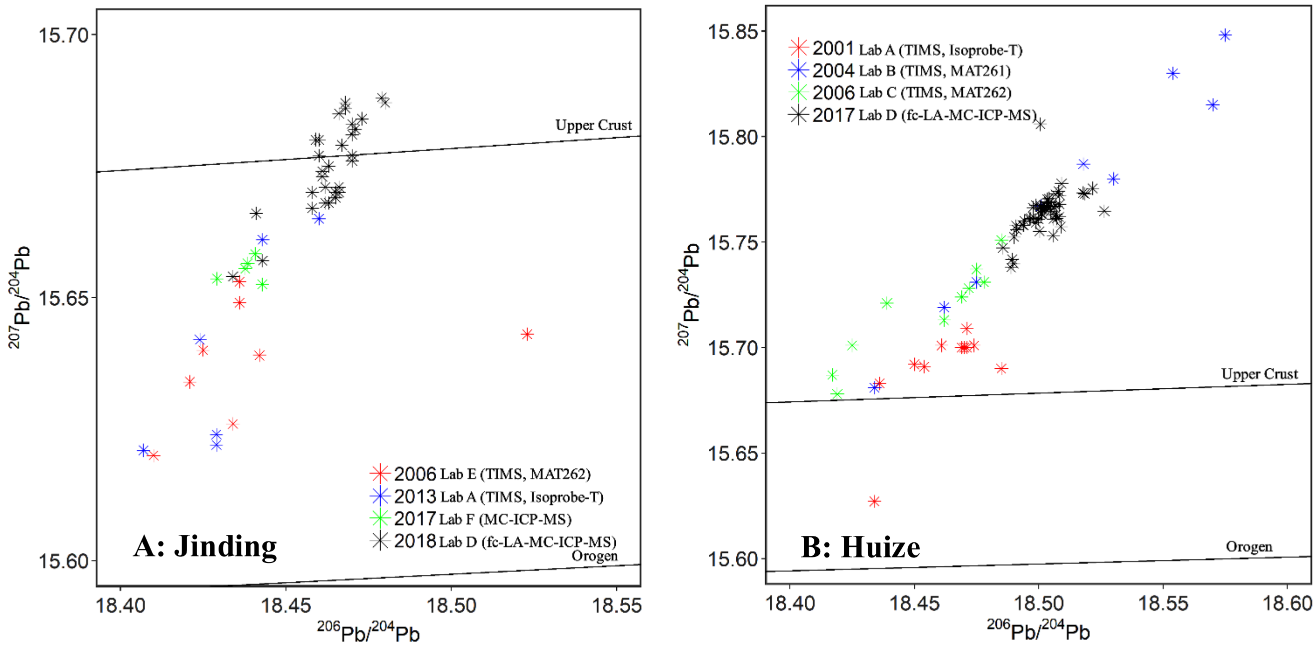

Figure 2. Comparison between isotopic values of galena samples between different laboratories. Lab A: Beijing Research Institute of Uranium Geology. Lab B: Yichang Institute of Geology and Mineral Resources, CAGS. Lab C: National Museum of Ethnology in Japan. D: State Key Laboratory of Continental Dynamics, Northwest University. E; Institute of Geology and Geophysics, CAS. F: Laboratory of Isotope Geology, MLR, Institute of Geology, CAGS.

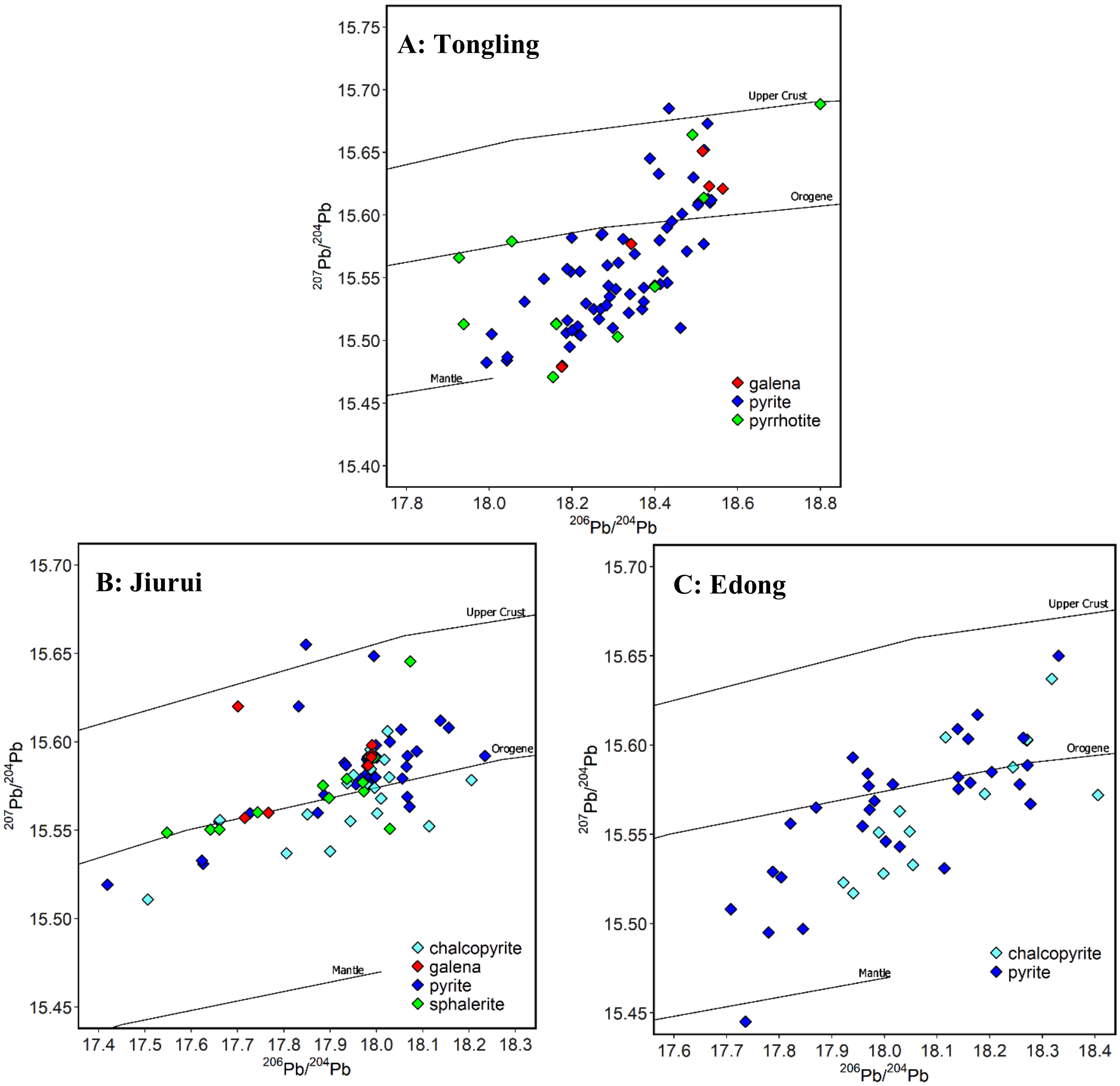

Figure 3. Comparison between isotopic values of sulfide samples between ore districts in the Middle-Lower Yangtze metallogenic belt. A: Tongling, B: Jiurui, and C: Edong.

References

1. Li Q and Bao ZW. Pb isotope constraints on the origin of the Jinding Zn-Pb deposit, Yunnan Province, China. Geochimica 2018; 47(2): 168–181 (In Chinese with English abstract).

2. Bao et al. Metal source of giant Huize Zn-Pb deposit in SW China: new constraints from in situ Pb isotopic compositions of galena. Ore Geology Reviews 2017; 91: 824–836

3. Ding TP, Yonge C, Schwarcz HP. Oxygen, hydrogen, and lead isotope studies of the Taolin lead-zinc ore deposit, China. Economic Geology 1986; 81: 421–429

4. Nie FJ and Bjorlykke A. Lead and sulfur isotope studies of the Wulashan Quartz-K Feldspar Gold deposit in Inner Mongolia. Economic Geology 1994; 89: 1289–1305

5. Zhang HF et al. Pb Isotopes suggest Devonian accretion of Yangtze (South China) craton to North China craton. Geology 1997; 25 (11): 1015–1018.

6. Wang YX et al. Geochemistry of granitoids from Zhejiang Province and crustal evolution: I. Phanerozoic granitoids. Geochimica 1997; 26 (5): 57–68 (In Chinese with English abstract).

7. Darling JR, Storey CD, Hawkesworth CJ. In-situ Pb isotope analysis of Fe-Ni-Cu sulphides by laser ablation multi-collector-ICPMS: new insights into ore formation in the Sudbury impact melt sheet. Geochim Cosmochim Acta 2012; 99: 1−17.

8. Liu ZF et al. Genesis of the Dongguashan skarn Cu-(Au) deposit in Tongling, Eastern China. Ore Geology Reviews 2019; 104: 462−476

9. Xu YM et al. Mineral chemistry and H–O–S–Pb isotopic compositions of skarn type copper deposits in the Jiurui district. Ore Geology Reviews 2015; 69: 88−103.

10. Lei XF et al. Ore-forming fluids and isotopic (H-O-C-S-Pb) characteristics of the Fujiashan-Longjiaoshan skarn W-Cu-(Mo) deposit in the Edong District. Ore Geology Reviews 2018; 102: 386−405.
