# Supplementary material for: A geochemical characterization of lead ores in China: An isotope database for provenancing archaeological materials
Source: PLoS One. 2019 Apr 24;14(4):e0215973. doi: 10.1371/journal.pone.0215973 (PMC6481871; doi:10.1371/journal.pone.0215973)
Supplement: S2 Appendix — (PDF) [file pone.0215973.s002.pdf]

S2 Appendix Contents of the Pb isotope database

Table 1. Database heading terms and abbreviations (*see* https://doi.org/10.7910/DVN/VID3WR)

**Column Headings**	**Definition**
ID	Identification numbers given for the sample data. Numbers 1-5594 are galena and feldspar, 5595-5864 are additional sulfide minerals for the Middle-Lower Yangtze, and 5865-5886 are ores from Jinshachang.
Sample No	Numbers given by the original publications
Specimen	Measured ore minerals
Site_En	English names for ore deposits, occurrence, or sampling location
Site_Cn	Chinese names for ore deposits, occurrence, or sampling location
Metal_Type	Economically profitable metals identified by the original publication
LAT	Lattitude location expressed in decimal degrees
LON	Longitute location expressed in decimal degrees
Metal_Province	Metallogenic province for ore deposits or sampling locations
Metal_District	Sub-unit of a metallogenic province
Metal_Field	Sub-unit of a metallogenic district
Metal_Sub_Field	Sub-unit of a metallogenic field
Admin_Province	Administrative province
206Pb/204Pb	$^{206}Pb/^{204}Pb$ isotopic ratio
207Pb/204Pb	$^{207}Pb/^{204}Pb$ isotopic ratio
208Pb/204Pb	$^{208}Pb/^{204}Pb$ isotopic ratio
Age	Model age calculated from Stacey and Kramer model (**see** Albaréde et al. 2012)
U/Pb	$^{238}U/^{204}Pb$ ratio calculated from Stacey and Kramer model (*see* Albaréde et al. 2012)
Th/U	$^{232}Th/^{238}U$ ratio calculated from Stacey and Kramer model (*see* Albaréde et al. 2012)
Conversion_Var	Variable for converting Pb isotopic ratio into relative Pb abundances
207Pb	Relative abundance of ^{207}Pb for ternary plotting
206Pb	Relative abundance of ^{206}Pb for ternary plotting
208Pb	Relative abundance of ^{208}Pb for ternary plotting
Reference	Publications where analyses were reported
Year	Year of publication
Quality	Analyses published after 2000 are marked as "Good" and before 2000 as "Bad"
Lab_Cn	Laboratory name in Chinese
Lab_En	Laboratory name in English
Analytical_Detail	Whether analytical details are provided by the authors or can be found elsewhere (Table 2)
Instrument	Instrument used for measurement

Table.2 List of Laboratories, instruments, and references for each data source

**Lab_Name**	**Instrument**	**Reference**
State Key Laboratory of Continental Dynamics, NWU	MC-ICP-MS (Nu Plasma HR) fs LA-MC-ICP-MS	Yuan et al. 2013. Determination of lead isotope compositions of geological samples using femtosecond laser ablation MC-ICPMS. Chin Sci Bull 58: 3914-3921<br><br>Dai et al. 2016. Simultaneous Measurement of Major, Trace Elements and Pb Isotopes in Silicate Glasses by Dai Laser Ablation Quadrupole and Multi-Collector Inductively Coupled Plasma Mass Spectrometry. Journal of Earth Science 28(1): 92–102
Australian Laboratory Services	HR-ICP-SFMS	余何等.2018.湘南江永铅锌矿床成因-来自S、Pb 同位素约束.矿产勘查 9(2): 213-222
Beijing Research Institute of Uranium Geology	Isoprobe-T MAT261	GBT 17672-1999 岩石中铅、锶、钕同位素测定方法<br><br>DZ/T0184.12-1997 岩石、矿物中微量铅的同位素组成的测定<br><br>Shen et al. 2012. Lead isotope geochemical characteristics of Pb-Zn-Cu deposits on the southwestern margin of Tarim, and their significance. Chin.J.Geochem 31:362–375<br><br>Zhou et al. 2001. The Source of Metals in the Qilinchang Zn-Pb Deposit, Northeastern Yunnan, China: Pb-Sr Isotope Constraints. Economic Geology 96: 583-598
Modern Analysis Center, Nanjing University	TIMS (VG354)	田世洪.2011.青海玉树东莫扎抓铅锌矿床S、Pb、Sr-Nd 同位素组成-对成矿物质来源的指示.岩石学报 27(7): 2173-2183
Guangzhou Institute of Geochemistry, CAS	МИ-1305 TIMS(VG354)	Zhu et al. 2001. The Pb, Sr and Nd isotopic features in organic matter from China and their implications for petroleum generation and migration. Geochim. Cosmochim. Acta 65, 2555–2570.<br><br>Zhang et al. 2001.Isotopic characteristics of shoshonitic rocks in eastern Qinghai-Tibet Plateau: Petrogenesis and its tectonic implication. SCIENCE IN CHINA (Series D) 44(1): 1-6
Institute of Geology and Geophysics, CAS	TIMS(VG354) TIMS(MAT262)	Zhang et al. 2002. Mesozoic lithosphere destruction beneath the North China Craton: evidence from major-, trace-element and Sr–

		Nd–Pb isotope studies of Fangcheng basalts. *Contrib Mineral Petrol* 144: 241–253
Laboratory of Isotope Geology, MLR, Institute of Geology, CAGS	MC-ICP-MS(Nu Plasma HR)	何学贤等 2005.多接收器等离子体质谱(MC-ICP-MS)Pb 同位素高精度研究.地球学报 26 (增刊): 19-22
State Key Laboratory of Geological Processes and Mineral Resources,<br>China University of Geosciences, Wuhan	MC-ICP-MS (Neptune Plus)<br>TIMS(MAT261)	Li.2016.Textures, trace elements, and Pb isotopes of sulfides from the Haopinggou vein deposit, southern North China<br><br>Zhang et al.1997.Pb Isotopes suggest Devonian accretion of Yangtze (South China) craton to North China craton
Commonwealth Scientific and Industrial<br>Research Organisation		Gulson. 1986. Lead isotope in mineral exploration. ELSEVIER, Amsterdam.
Wuhan Institute of Geology and Mineral Resources<br>Yichang Institute of Geology and Mineral Resources, CAGS	TIMS(MAT261)	DZ/T0184.12-1997 岩石、矿物中微量铅的同位素组成的测定<br><br>Qian et al. 2002. An estimate of the lead isotopic compositions of upper mantle and upper crust and implications for the source of lead in the Jinding Pb-Zn deposit in Western Yunnan, China. Geochemical Journal 36: 271-282
Guilin Institute of Geology for Mineral Resources	TIMS(MAT261)	王谦等.2011.湖南柿竹园矿床 Pb 同位素地球化学特征.礦物學報增刊: 645-646
Department of Earth and Atmospheric Sciences, University of Alberta<br>Mineralogisk-Geologisk Museum, Oslo	VGM30	Birkeland, A. 1990: Pb-isotope analysis of sulphides and K-feldspars. A short introduction to<br>analytical techniques and evaluation of results. INTERN SKRIFTSERIE Nr. 15. Mineralogisk-Geologisk Museum, Oslo.<br><br>Birkeland, A. & Bjørlykke, A.: Pb isotopic constraints on the origin of the Husvika Zn-Pb deposit in Nordland, north-central Norway. Norsk Geologisk Tidsskrift 73: 43-54
State Key Laboratory for Mineral Deposit Research<br>, Nanjing University	Triton TI	侯明兰.2006.胶东蓬莱金成矿区的 S-Pb 同位素地球化学和 Rb-Sr 同位素年代学研究.岩石學報 22(10): 2525-2533
Laboratory for Chemical Geodynamics, University of Science	TIMS(MAT262)	Chen et al. 2005. Pb isotope geochemistry of lead, zinc, gold, and silver deposit clustered regions, Liaodong rift zone. Science in China Ser. D Earth Sciences 48(4): 467-476

and Technology of China.		
U.S. Geological Survey		Church. 2010.Lead Isotope Database of Unpublished Results from Sulfide Mineral Occurrences—California, Idaho, Oregon, and Washington
