# Supplementary material for: A geochemical characterization of lead ores in China: An isotope database for provenancing archaeological materials
Source: PLoS One. 2019 Apr 24;14(4):e0215973. doi: 10.1371/journal.pone.0215973 (PMC6481871; doi:10.1371/journal.pone.0215973)
Supplement: S3 Appendix — (PDF) [file pone.0215973.s003.pdf]

S3 Appendix Bivariate diagrams for Fig 11–Fig 13

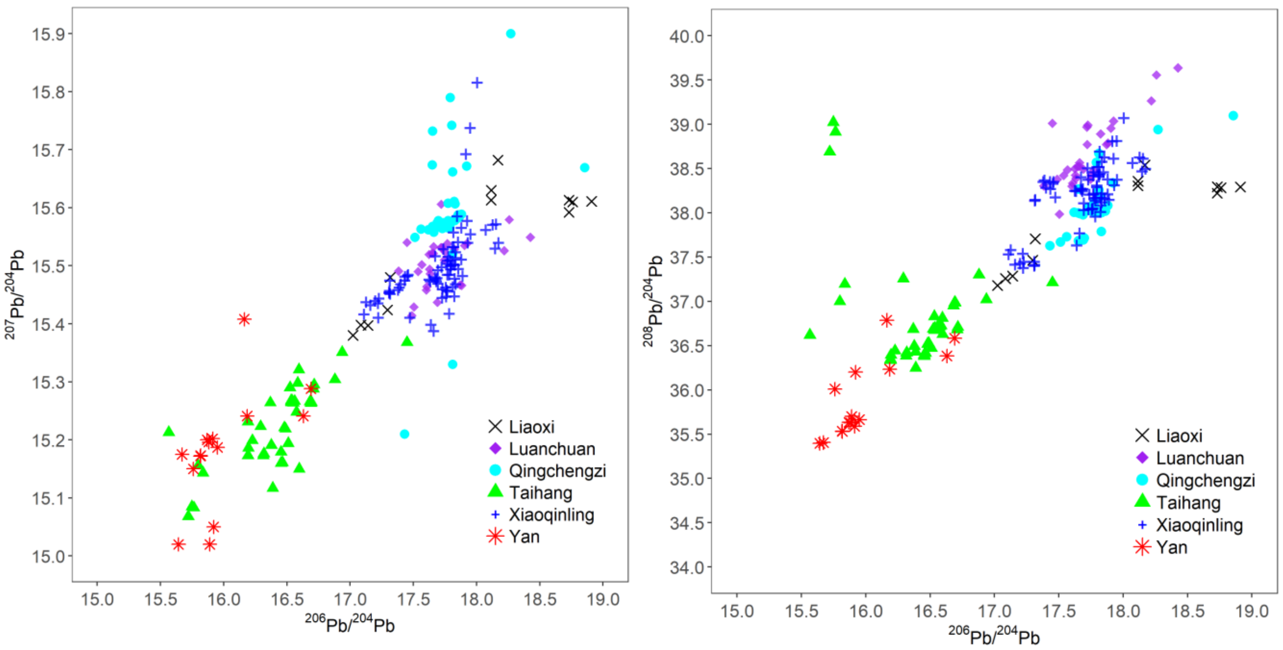

Bivariate Plots Corresponding to Figure 11A

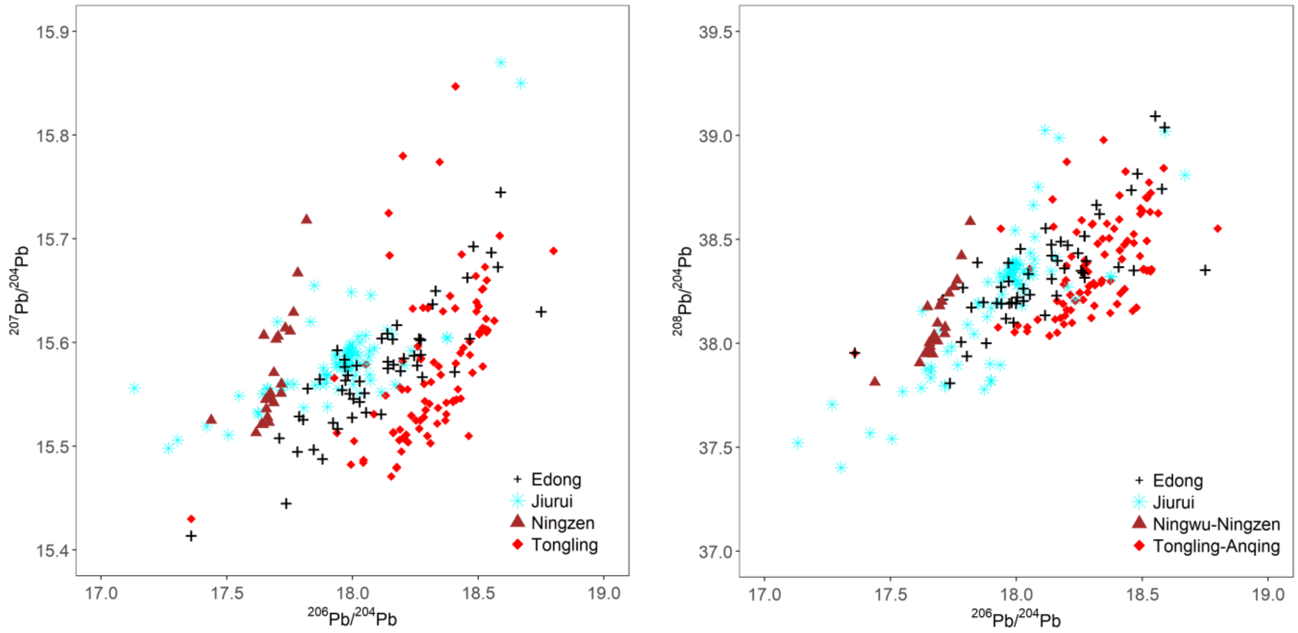

Bivariate Plots Corresponding to Figure 11B

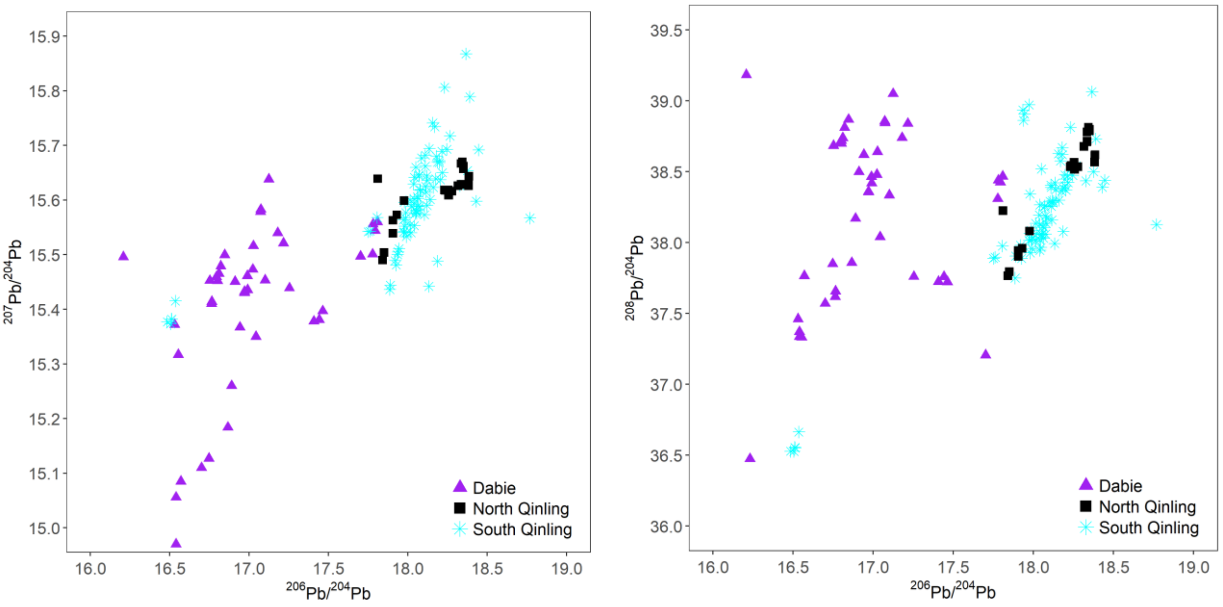

Bivariate Plots Corresponding to Figure 11C

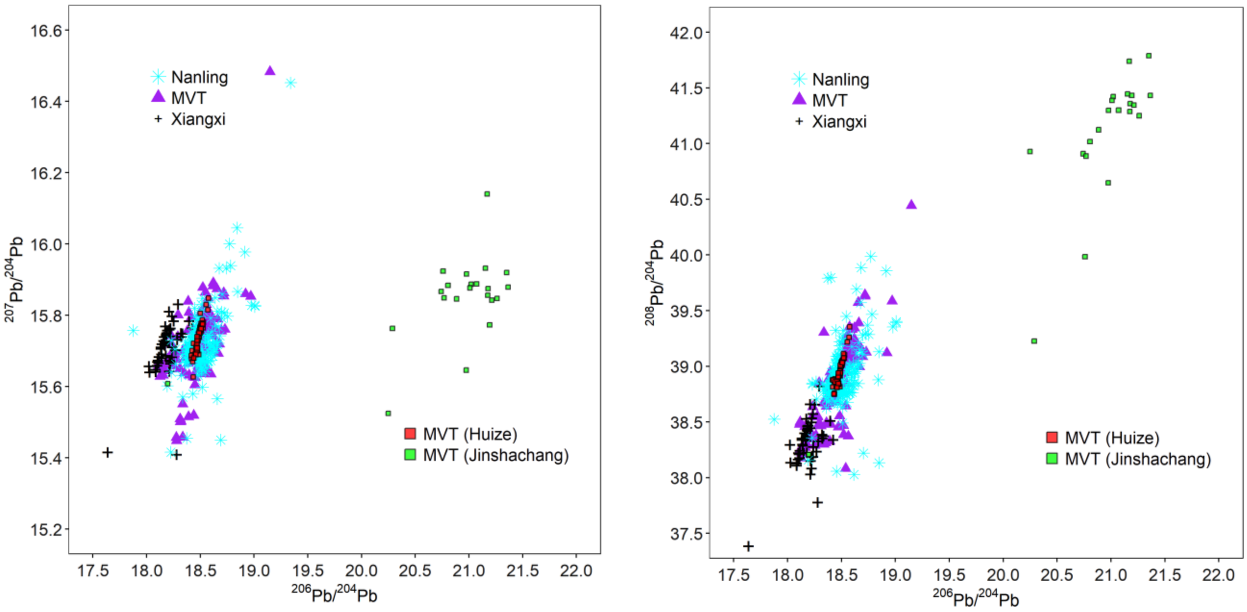

Bivariate Plots Corresponding to Figure 12B

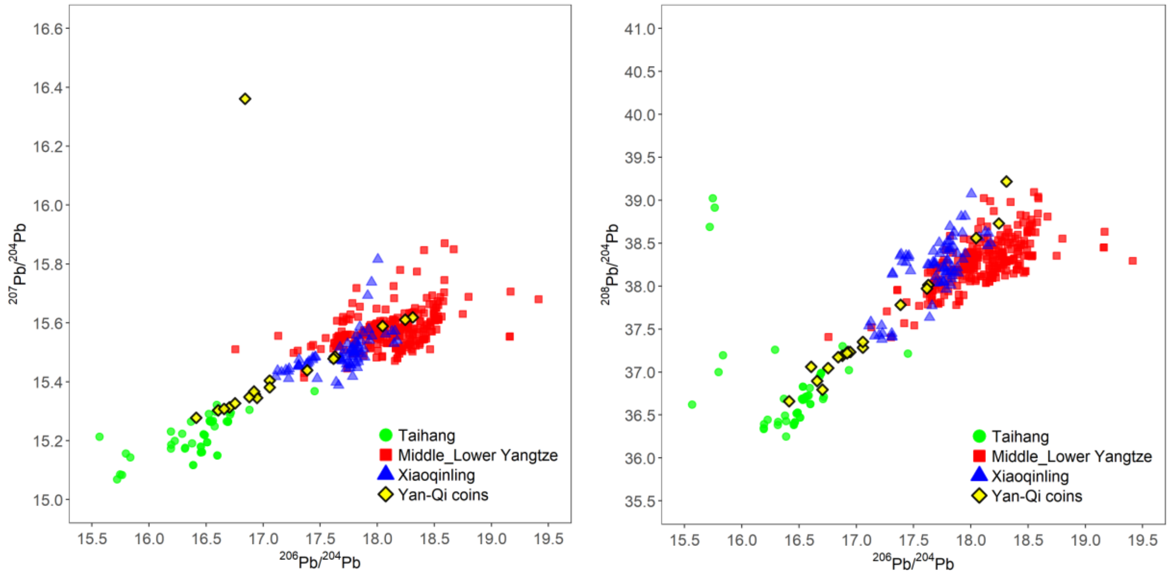

Bivariate Plots Corresponding to Figure 13A

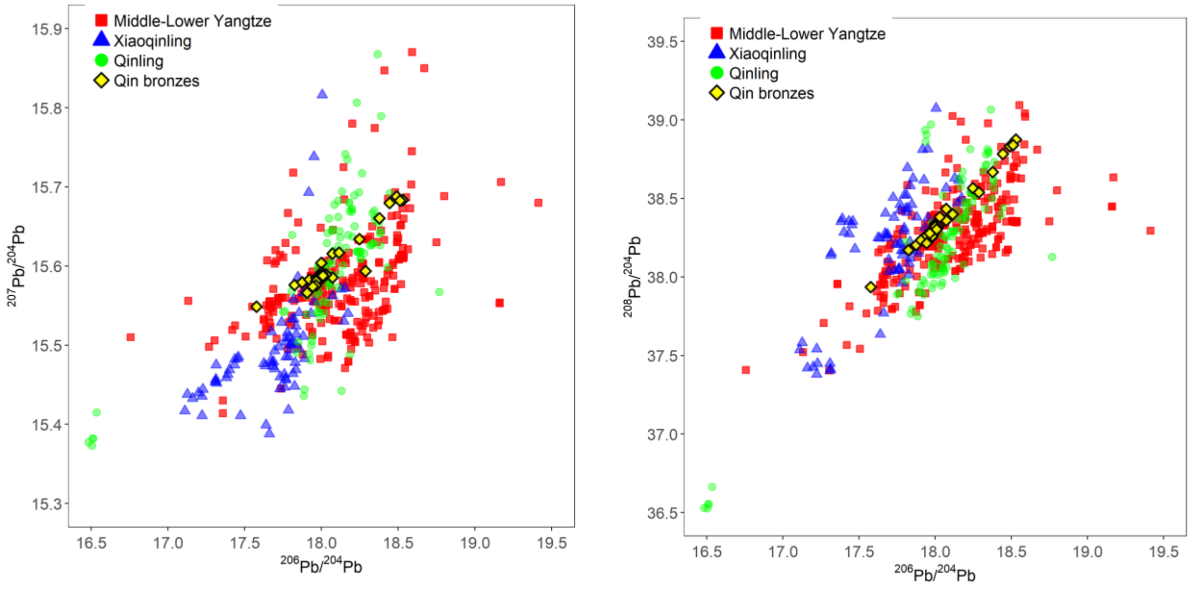

Bivariate Plots Corresponding to Figure 13B

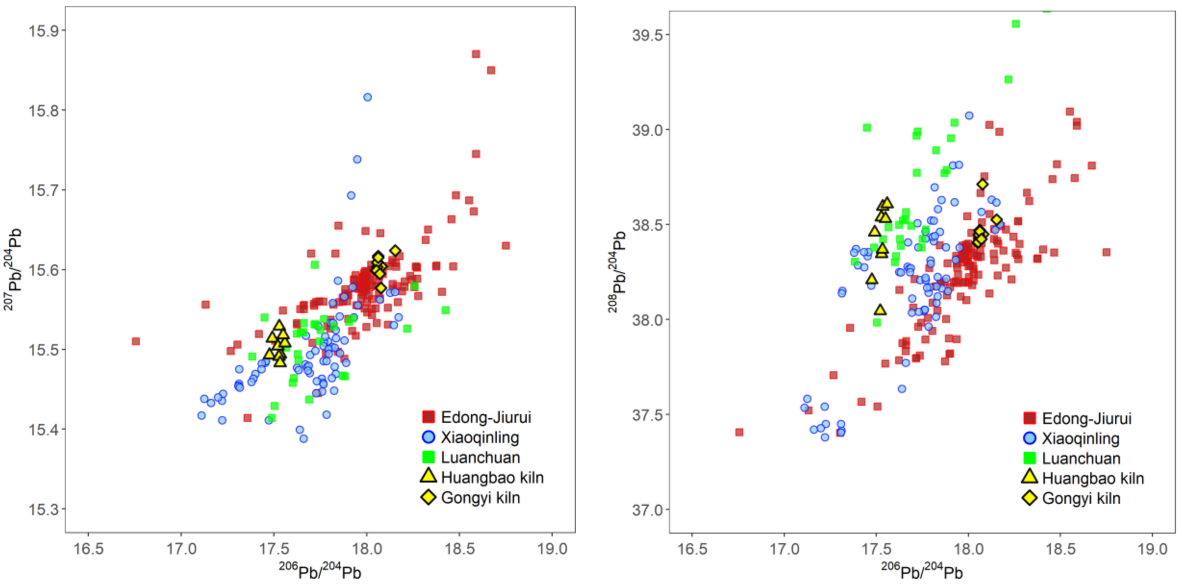

Bivariate Plots Corresponding to Figure 13C
